# The prevalence of psychological disorders among cancer patients during the COVID‐19 pandemic: A meta‐analysis

**DOI:** 10.1002/pon.6012

**Published:** 2022-08-19

**Authors:** Lemeng Zhang, Xiaohong Liu, Fei Tong, Ran Zhou, Wanglian Peng, Hui Yang, Feng Liu, Desong Yang, Xufen Huang, Minni Wen, Ling Jiang, Lili Yi

**Affiliations:** ^1^ Thoracic Medicine Department 1 Hunan Cancer Hospital/The Affiliated Cancer Hospital of Xiangya School of Medicine Central South University Changsha Hunan China; ^2^ Department of Clinical Spiritual Care Hunan Cancer Hospital/The Affiliated Cancer Hospital of Xiangya School of Medicine Central South University Changsha Hunan China; ^3^ Department of Radiation Oncology Hunan Cancer Hospital/The Affiliated Cancer Hospital of Xiangya School of Medicine Central South University Changsha Hunan China; ^4^ The Second Department of Thoracic Surgery Hunan Cancer Hospital/The Affiliated Cancer Hospital of Xiangya School of Medicine Central South University Changsha Hunan China

**Keywords:** anxiety, cancer, COVID‐19, depression, distress, fear of cancer progression/recurrence, oncology, PTSD

## Abstract

**Purpose:**

We aimed to assess the prevalence rate (PR) of depression, anxiety, posttraumatic stress disorder (PTSD), insomnia, distress, and fear of cancer progression/recurrence among patients with cancer during the COVID‐19 pandemic.

**Methods:**

Studies that reported the PR of six psychological disorders among cancer patients during the COVID‐19 pandemic were searched in PubMed, Embase, PsycINFO, and Web of Science databases, from January 2020 up to 31 January 2022. Meta‐analysis results were merged using PR and 95% confidence intervals, and heterogeneity among studies was evaluated using *I* ^*2*^ and Cochran's Q test. Publication bias was examined using funnel plots and Egger's tests. All data analyses were performed using Stata14.0 software.

**Results:**

Forty studies with 27,590 participants were included. Pooled results showed that the PR of clinically significant depression, anxiety, PTSD, distress, insomnia, and fear of cancer progression/recurrence among cancer patients were 32.5%, 31.3%, 28.2%, 53.9%, 23.2%, and 67.4%, respectively. Subgroup analysis revealed that patients with head and neck cancer had the highest PR of clinically significant depression (74.6%) and anxiety (92.3%) symptoms. Stratified analysis revealed that patients with higher education levels had higher levels of clinically significant depression (37.2%). A higher level of clinically significant PTSD was observed in employed patients (47.4%) or female with cancer (27.9%).

**Conclusion:**

This meta‐analysis evaluated the psychological disorders of cancer patients during the COVID‐19 outbreak. Therefore, it is necessary to develop psychological interventions to improve the mental health of cancer patients during the pandemic.

## 1 INTRODUCTION

The coronavirus disease 2019 (COVID‐19) pandemic poses a serious threat to public health globally and is a time of unprecedented psychosocial disorder for many people.^1^, ^2^ Due to the uncertainty of COVID‐19, the psychology and spirit of individuals have been affected.^3^ People are worried about becoming infected and how long the pandemic will last. Dong et al.^4^ reported potential causes for the increased psychological problems in the general population during this pandemic. The virus has an uncertain incubation and may be accompanied by asymptomatic transmission, causing additional anxiety and fear among the public. The World Health Organization (WHO) declared that the prevalence of psychological disorder in the general population has risen dramatically worldwide and will become a global burden.^5^

Psychological disorder is common in cancer patients and is associated with poor health outcomes,^6^ and approximately 30%–50% of cancer patients suffer from psychological distress.^7^ Owing to the immunosuppression induced by cancer and the treatment, patients with cancer are more susceptible to COVID‐19 infection; meanwhile, cancer patients may exhibit a higher risk of death if they infected.^8^ In addition, the reduction in services and delayed or missed counseling and treatment due to the shortage of medical resources has further adverse impacts on the mental health of cancer patients.^9^, ^10^ Meanwhile, loneliness caused by social distancing further affect the emotional well‐being.^11^ Evidence suggests that the COVID‐19 pandemic exacerbates the psychological disorder of cancer patients.^12^ Bargon^13^ compared the psychological states of breast cancer patients and survivors before and during the outbreak and found that emotional functioning deteriorated in these patients, and loneliness increased in nearly half of them. Taken together, illness and COVID‐19 are dual challenges for cancer patients. Maintaining a stable psychological state is key to ensuring that patients can receive effective cancer treatment. Given the vulnerability of cancer patients to psychological disorders, their psychological changes during COVID‐19 infection need to receive increased attention from caregivers and social organizations.^14^

Individuals affected by this pandemic may experience psychological distress, such as anxiety, stress, depression, insomnia, and suicidal behavior.^15^ A previously published meta‐analysis revealed the prevalence of depression and anxiety among patients with cancer during the COVID‐19 outbreak.^16^ However, the impact of COVID‐19 on other psychological disorder in cancer patients has not been systematically studied. Therefore, we performed an updated meta‐analysis to determine the prevalence rate (PR) of psychological disorders in cancer patients during the COVID‐19 outbreak, including anxiety, depression, posttraumatic stress disorder (PTSD), insomnia, distress, and fear of cancer progression/recurrence. Our research will help support the development of policy interventions to mitigate psychological issues among cancer patients during COVID‐19 pandemic.

## 2 METHODS

This meta‐analysis was performed in accordance with the Preferred Reporting Items for Meta‐Analyses (PRISMA) guidelines and was registered with PROSPERO (CRD42022308459). All analyses were based on previous published studies, thus no ethical approval and patient consent are required for this meta‐analysis.

### 2.1 Data source and retrieval strategy

Candidate studies were searched from PubMed, Embase, PsycINFO, and Web of Science databases from 1 January 2020 and 31 January 2022, without language restrictions. The following search algorithms were applied: (“depression” OR “distress” OR “stress” OR “anxiety” OR “post‐traumatic stress symptoms” OR “post‐traumatic stress disorder” OR “burnout” OR “psychological”) AND (“neoplasm” OR “cancer” OR “tumor” OR “tumour”) AND (“COVID–19” OR “SARS‐CoV–2” OR “severe acute respiratory syndrome coronavirus 2”). The retrieval strategy was adjusted according to the characteristics of each database (Tables S1–S4). Further, to obtain more potential studies that could be used for meta‐analysis, we manually searched the literature and reference lists of relevant reviews and included studies.

### 2.2 Selection criteria

The following criteria for inclusion were applied: (1) cancer patients were pathologically diagnosed or treated in the hospital; (2) or studies reported the PR of at least one mental health outcomes: anxiety, depression, PTSD, insomnia, distress, and fear of cancer progression/recurrence during COVID‐19 pandemic; (3) the cut‐off value for the mental health status of patients evaluated by the Patient Health Questionnaire‐9 (PHQ‐9), Impact of Event Scale‐Revised (IES‐R), Insomnia Severity Index (ISI), and other scales was reported; and (4) research designs were cross‐sectional or cohort studies.

The exclusion criteria were as follows: (1) mental status scores in patients with cancer were reported as mean ± SD, not PR, and (2) non‐treatise literature such as letters, reviews, and comments. Furthermore, for duplicate publications or multiple articles with the same data, we only included articles with the most complete information.

### 2.3 Data extraction and quality assessment

Two researchers independently extracted information from each study, including the first author, publication year, research area, socio‐demographic information (gender, age, sample size, marital status, education, and employment status), study type, type and stage of cancer, measurement scales, and cut‐off value. After data extraction, inconsistencies were resolved through discussion.

The Joanna Briggs Institute (JBI), containing nine items, was used to perform a bias risk assessment.^17^ Each item can be judged as “yes,” “no,” and “unclear or not applicable,” corresponding to “low risk,” “high risk,” and “unclear risk.” In brief, a study with at least one item at “high risk” is defined as “high” risk of bias; studies with at least three items at “unclear risk” are defined as “unclear” risk of bias; the remaining studies are regarded as “low” risk of bias.

### 2.4 Statistical analysis

The prevalence of each mental health outcome in cancer patients was evaluated using PR with a 95% confidence interval (CI). *P* < 0.05 and/or *I* ^*2*^ > 50% represented significant heterogeneity between studies, and a random‐effects model was used to merge the effect size. *p* ≥ 0.05 and *I* ^*2*^ ≤ 50% represented no heterogeneity, and a fixed‐effects model was applied. To explore the source of heterogeneity, a subgroup analysis was conducted according to several variables (area, scale, risk of bias, cancer type, gender, marital status, education level, and employment status). Publication bias was examined using funnel plots and Egger's tests.^18^ All meta‐analyses were performed using the Stata14.0 software (Stata Corp, College Station).

## 3 RESULTS

### 3.1 Study selection

**FIGURE 1:**
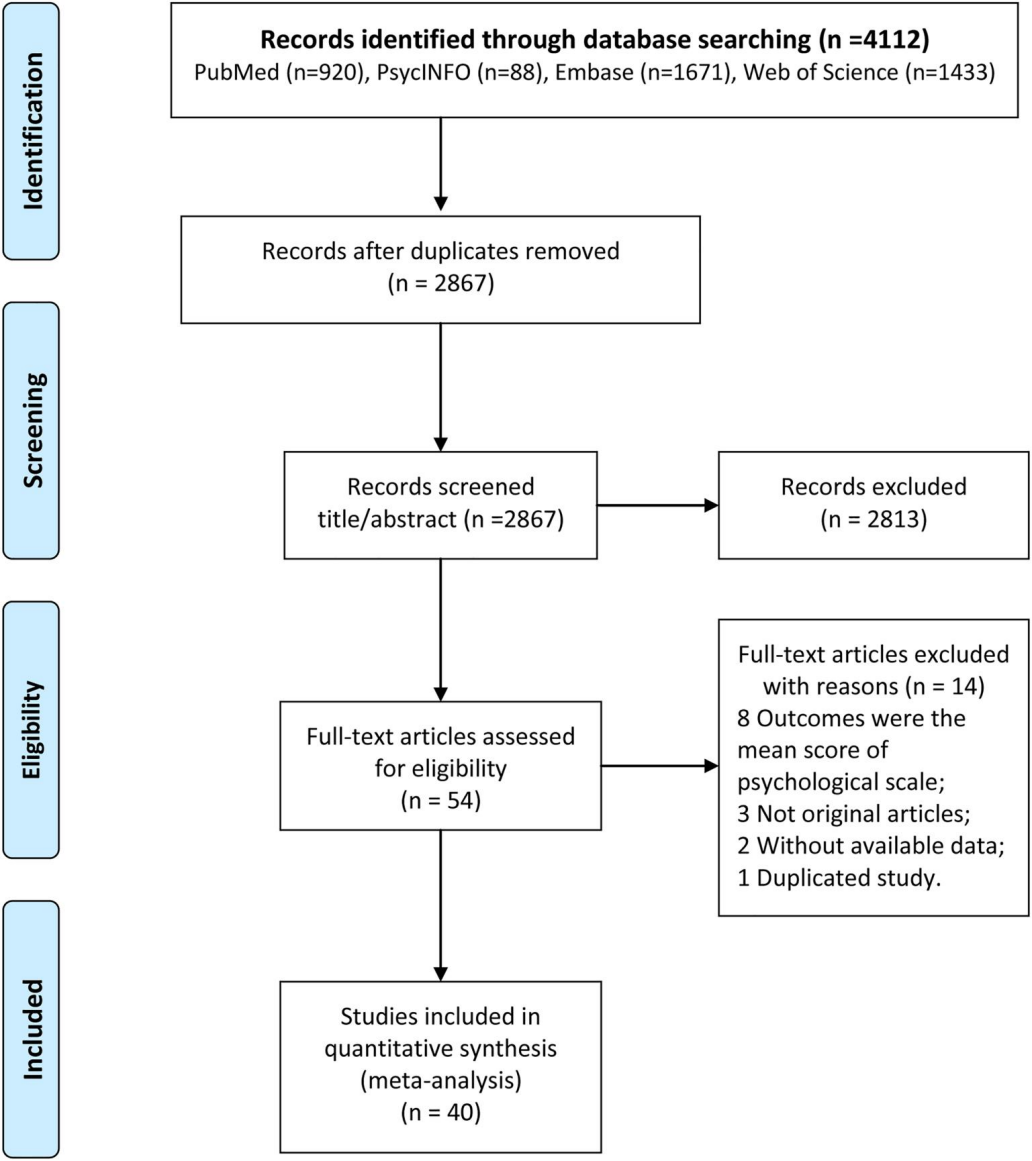
Flowchart of study selection according to the PRISMA guidelines

A flowchart of the search results is shown in Figure 1. A total of 920, 1,671, 88, and 1433 articles were preliminarily screened in the PubMed, Embase, PsycINFO, and Web of Science databases, respectively. Then, 2867 duplicate records and 2867 articles that did not meet the inclusion criteria were deleted. Finally, 40 studies were included in this meta‐analysis. The summarized psychological health status is presented in Table S5.

### 3.2 Characteristics of each included study

The detailed characteristics of the 40 included studies are presented in Table 1. Among these, although four studies^20^, ^35^, ^44^, ^49^ were cohort studies, this study only extracted data at a certain time point for meta‐analysis. These studies were conducted in China, Italy, the Netherlands, America, Canada, and other countries. A total of 27,590 cases were included, and the sample size of each study ranged from 14 to 6231 participants. As for risk of bias, 11,^24^, ^27^, ^29^, ^31^, ^34^, ^37^, ^38^, ^40^, ^47^, ^50^, ^56^ 14,^23^, ^25^, ^28^, ^32^, ^33^, ^35^, ^41^, ^42^, ^43^, ^44^, ^49^, ^52^, ^54^, ^55^ and 15^19^, ^20^, ^21^, ^22^, ^26^, ^30^, ^36^, ^39^, ^45^, ^46^, ^48^, ^51^, ^53^ studies had high, unclear, and low risks, respectively (Table S6). Overall, the risk of bias for the included studies was relatively small, indicating moderate methodological quality.

**TABLE 1:** Characteristics of 40 included studies in this meta‐analysis

Study	Area	Type of study	*n*, *M*/*F*	Age, years	Married/Unmarried	Education level, highschool or below/University or above	Employed/Unemployed	Type of cancer	Stage of cancer
Alrubai, T 2021^19^	Iraq	CSS	200, 17/183	22 16–39years; 139 40–64years; 39 ≥ 65years	159/41	101/99	73/127	Mixed	Non‐metastatic
Arrieta, O 2021^20^	Mexico	PCS	144, NR	61.5 ± 12.9	NR	NR	NR	Thoracic	10 I, 17 II, 60 III, 442 IV, 19 missing
Bafunno, D 2021^21^	Italy	CSS	178, 87/91	58 ± 14.4	NR	115/35	53/101	Mixed	NR
Bao, M 2021^22^	China	CSS	3197, 1831/1366	43 (16–92)^a^	2486/711	1798/1399	NR	Hematological	NR
Bauerle, A 2021^23^	Germany	CSS	150, 72/78	17 < 45years; 122 45–74years; 11 ≥ 75years	110/40	55/95	47/103	Mixed	10 I, 11 II, 21 III, 36 IV, 72 missing
Borsari, S 2021^24^	Italy	CSS	355, 161/165	56.3 (17.3) ^b^	NR	269/79	140/207	Skin	NR
Chen, GL 2020^25^	China	CSS	326, 174/152	201 18–60; 125 > 60	278/48	218/108	NR	Mixed	NR
Chen, X 2021^26^	China	CSS	834, 0/834	291 < 46years; 543 ≥ 46years	717/117	521/313	NR	Breast	757 early or middle stage, 77 late stage
Ellehuus, C 2021^27^	Denmark	CSS	2239, 1268/966	67 ± 13.3	1612/625, 2 missing	861/1171	NR	Hematological	NR
Faro, JM 2021^28^	USA	CSS	61, 10/51	62 ± 10.4	38/23	4/57	NR	NR	NR
Forner, D 2021^29^	Canada	CSS	14, 4/10	59 ± 10.6	8/6	4/10	NR	Mixed	NR
Frey, MK 2020^30^	USA	CSS	555, 0/555	58 (20–85)^a^	NR	NR	NR	Gynaecological	170 I‐II, 321 III‐IV, 14 missing
Guc, ZG 2021^31^	Turkey	CSS	761, 281/480	58 ± 11.67	NR	695/66	183/578	Mixed	354 local, 31 local advanced, 376 metastatic
Gultekin, M 2021^32^	Europe	CSS	1251, 0/1251	55 (18–89)^a^	NR	NR	NR	Gynaecological	NR
Hu, L 2020^33^	China	CSS	156, 81/75	7 < 30years; 149 ≥ 30years	143/13	105 < 9years; 51 ≥ 9years	135/21	Mixed	29 I‐II, 127 III‐IV
Jacobson, C 2021^34^	UK	CSS	112, 37/71	16–30	NR	NR	NR	Mixed	NR
Joly, F 2021^35^	France	PCS	563, 154/409	417 < 70years; 146 ≥ 70years	NR	NR	NR	Mixed	NR
Juanjuan, L 2020^36^	China	CSS	658, 0/658	152 < 40years; 364 40–54years; 123 55–64years; 19 > 64years	584/74	515/143	NR	Breast	392 early, 115 metastatic, 151 missing
Kamposioras, K 2020^37^	UK	CSS	143, 115/25	11 31–40years; 14 41–50years; 29 51–60years; 44 61–70years; 27 71–75years; 17 > 75years	NR	NR	NR	Colorectal	NR
Levy, I 2021^38^	Israel	CSS	408, 192/216	60 ± 14	322/86	98/307	153/254	Hematological	NR
Lou, SC 2020^39^	China	CSS	58, 35/23	46 ± 8.3	47/11	31/27	NR	Head and neck	II‐IV
Massicotte, V 2021^40^	Canada	CSS	36, 0/36	53.6 ± 10.9	24/12	10/26	9/27	Breast	Non‐metastatic
Mendonca, AB 2021^41^	Brazil	CSS	91, 41/50	55.4 ± 13.9	NR	73/18	NR	Mixed	2 I, 11 II, 37 III, 38 IV, 3 missing
Nardone, V 2021^42^	Italy	CSS	78, 39/39	13 < 50years; 58 50–70years; 7 > 70years	NR	NR	NR	Mixed	NR
Ng, DWL 2020^43^	HongKong	CSS	72, 0/72	52.96 ± 8.34	46/26	8 < 9years; 64 ≥ 9years	36/36	Breast	NR
Ng, KYY 2020^36^	Singapore	CSS	624, 239/349	57.2 ± 12.2	511/107, 6 missing	439/173, 12 missing	296/315, 13 missing	Mixed	39 I, 55 II, 86 III, 177 IV, 267 missing
Rades, D 2020^44^	Germany	RCS	338, 0/338	169 < 61yearears; 169 ≥ 61year	NR	NR	NR	Breast	NR
Rodrigues‐Oliveira, L 2021^45^	Brazil	CSS	50, 39/11	58.8 ± 9.89	27/23	45/5	25/25	Head and neck	1 I, 1 II, 15 III, 33 IV
Romito, F 2020^46^	Italy	CSS	77, 39/38	56.6 (22–85)^a^	NR	57/17, 3 missing	32/42, 3 missing	Hematological	NR
Soriano, EC 2021^47^	USA	CSS	50, 0/50	60.1 ± 13.2	NR	21/29	32/18	Breast	0–III
Toquero, P 2021^48^	Spain	CSS	104, 37/67	60 < 65years; 44 ≥ 65years	56/48	64/40	NR	Mixed	38 localized, 66 metastatic
Turgeman, I 2021^49^	Israel	PCS	164, 72/92	23–90	NR	NR	NR	Mixed	66 localized, 98 metastatic
van de Poll‐Franse, LV 2021^50^	Netherlands	CSS	4094, 2493/1601	63.0 ± 11.1	NR	NR	NR	NR	NR
Wang, Y 2020^51^	China	CSS	6213, 3278/2935	50.57 ± 13.28	5452/761	3777/2436	2553/3660	Mixed	NR
Wong, LP 2021^52^	Malaysia	CSS	631, 147/457	160 21–49years; 230 50–60years; 241 61–86years	NR	225/406	NR	Mixed	41 0, 190 I, 246 II, 123 III, 31 IV
Yang, L 2021^53^	China	CSS	373, 245/128	57 (22–89)^a^	348/25	314/39	129/244	Mixed	14 I, 82 II, 158 III, 119 IV
Yang, SJ 2021^54^	China	CSS	219, 51/168	102 ≤ 40years; 117 > 40years	184/33	47/172	159/60	Thyroid	NR
Yang, SL 2021^55^	China	CSS	609, NR	NR	NR	NR	NR	NR	NR
Yang, SM 2020^53^	China	CSS	1106, 482/624	24 18–20years; 495 20–39years; 477 40–59years; 108 60–79years; 2 ≥ 80years	NR	356/750	NR	Hematological	416 early, 519 advanced, 99 missing
Yasin, AI 2021^56^	Turkey	CSS	298, 0/298	53.2 ± 10.79	233/65	NR	19/277	Breast	231 localized, 67 metastatic

Abbreviations: CSS, cross‐sectional study; F, female; M, male; NR, not reported; PCS, prospective cohort study; PCS, retrospective cohort study; UK, United Kingdom; USA, United States of America.

^a^ median (range).

^b^ median (IQR).

### 3.3 Results of meta‐analysis and subgroup analysis

#### 3.3.1 Depression

**FIGURE 2:**
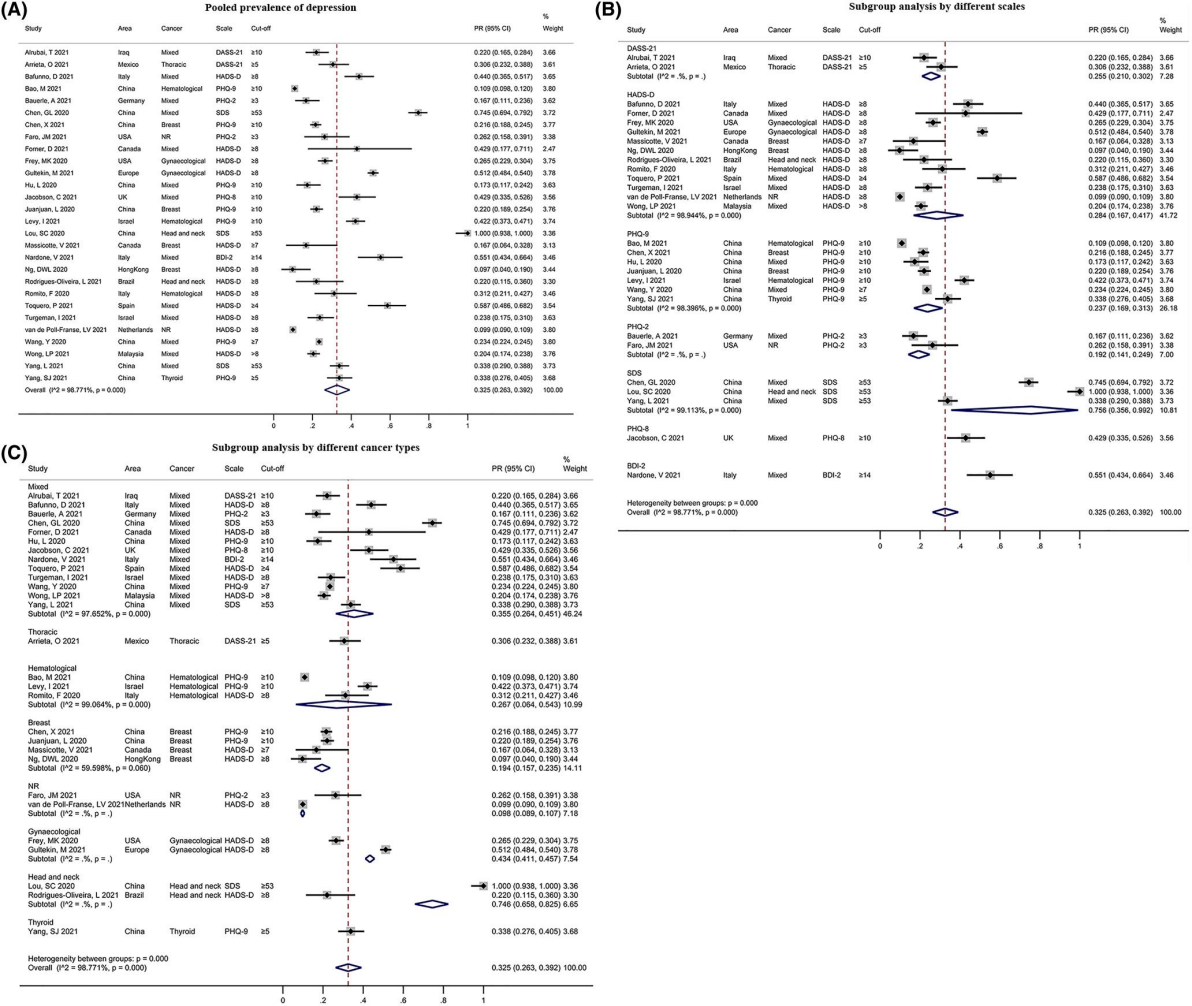
Forest plot of the prevalence rate (PR) of depression among patients with cancer. (A) Pooled PR of depression. (B) Subgroup analysis of the PR of depression based on different scales. (C) Subgroup analysis of the PR of depression based on different cancer types

A total of 28 studies reported the PR of depression among cancer patients. The pooled result was 32.5% (95% CI: 0.263, 0.392, Figure 2A), with a significant heterogeneity (*I* ^*2*^ = 98.771%, *P* < 0.001). In a subgroup analysis, there were significant differences among different evaluation scales (*P* < 0.01, Figure 2B), and the PR of depression ranged from 19.2% (95% CI: 0.141, 0.249) to 75.6% (95% CI: 0.356, 0.992). Moreover, significant differences among patients with different types of cancer were observed (*P* < 0.01, Figure 2C). Among these, patients with head and neck cancer had the highest PR for depression (74.6%, 95% CI: 0.658, 0.825). However, we found no statistically significant differences in the subgroup analysis by area or risk of bias (*P* > 0.05, Figure S1A and S1B).

#### 3.3.2 Anxiety

**FIGURE 3:**
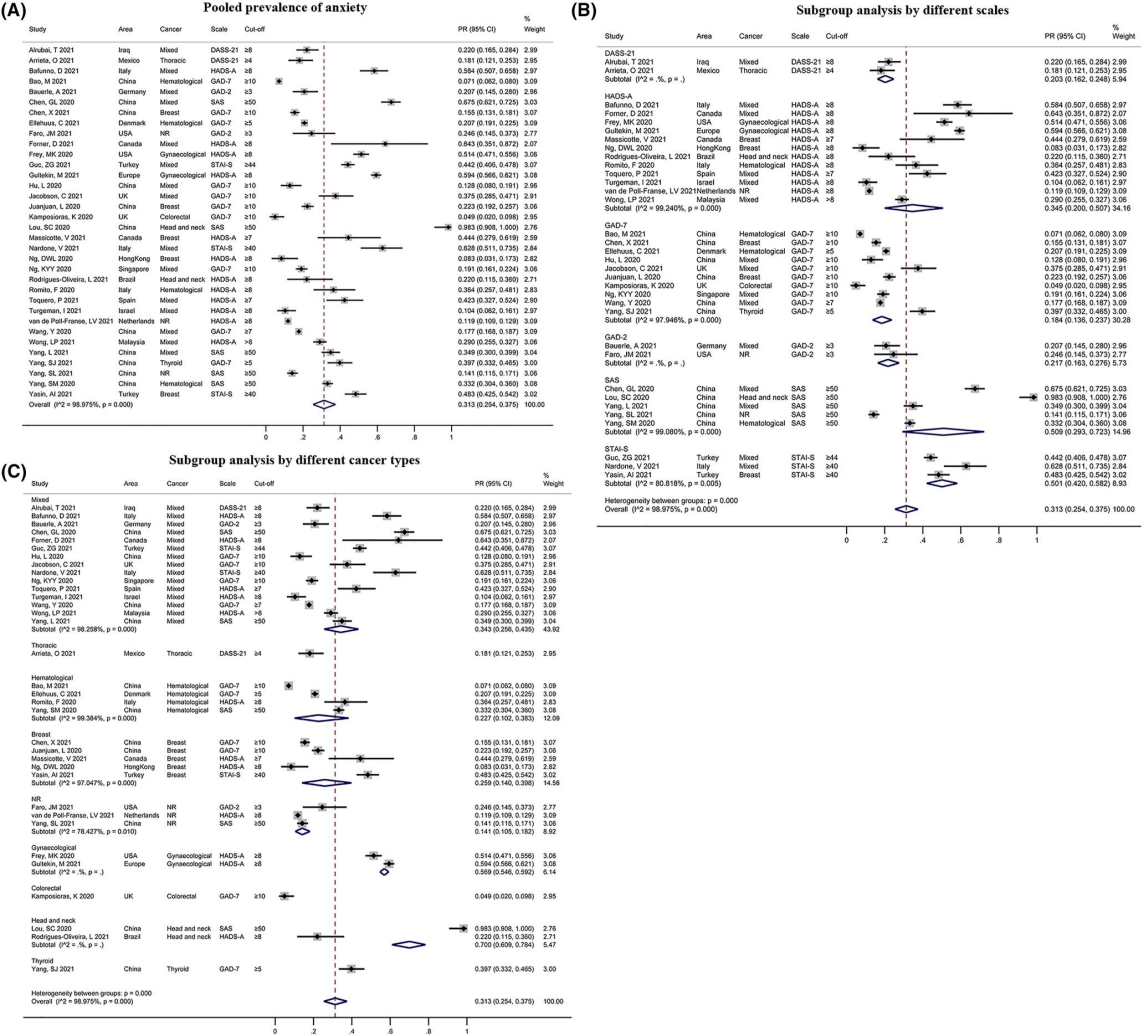
Forest plot of the PR of anxiety among patients with cancer. (A) Pooled PR of anxiety. (B) Subgroup analysis of the PR of anxiety based on different scales. (C) Subgroup analysis of the PR of anxiety based on different cancer types

A total of 34 studies reported the anxiety prevalence for cancer patients; the pooled PR of anxiety was 31.3% (95%: 0.254, 0.375, Figure 3A), and the heterogeneity among studies was significant (*I* ^*2*^ = 98.975%, *P* < 0.001). Significant results were found in the subgroup analysis using different scales (*P* < 0.01, Figure 3B), revealing that the PR of anxiety ranged from 18.4% (95% CI: 0.136, 0.237) to 50.9% (95% CI: 0.293, 0.723). Moreover, the PR of anxiety varied significantly among patients with different types of cancer (*P* < 0.01, Figure 3C), and patients with head and neck cancer were associated with the highest rate of anxiety (92.3%, 95% CI: 0.891, 0.955). However, subgroup analyses by area and risk of bias were not significant (*P* > 0.05; Figures S2A and S2B).

#### 3.3.3 PTSD

**FIGURE 4:**
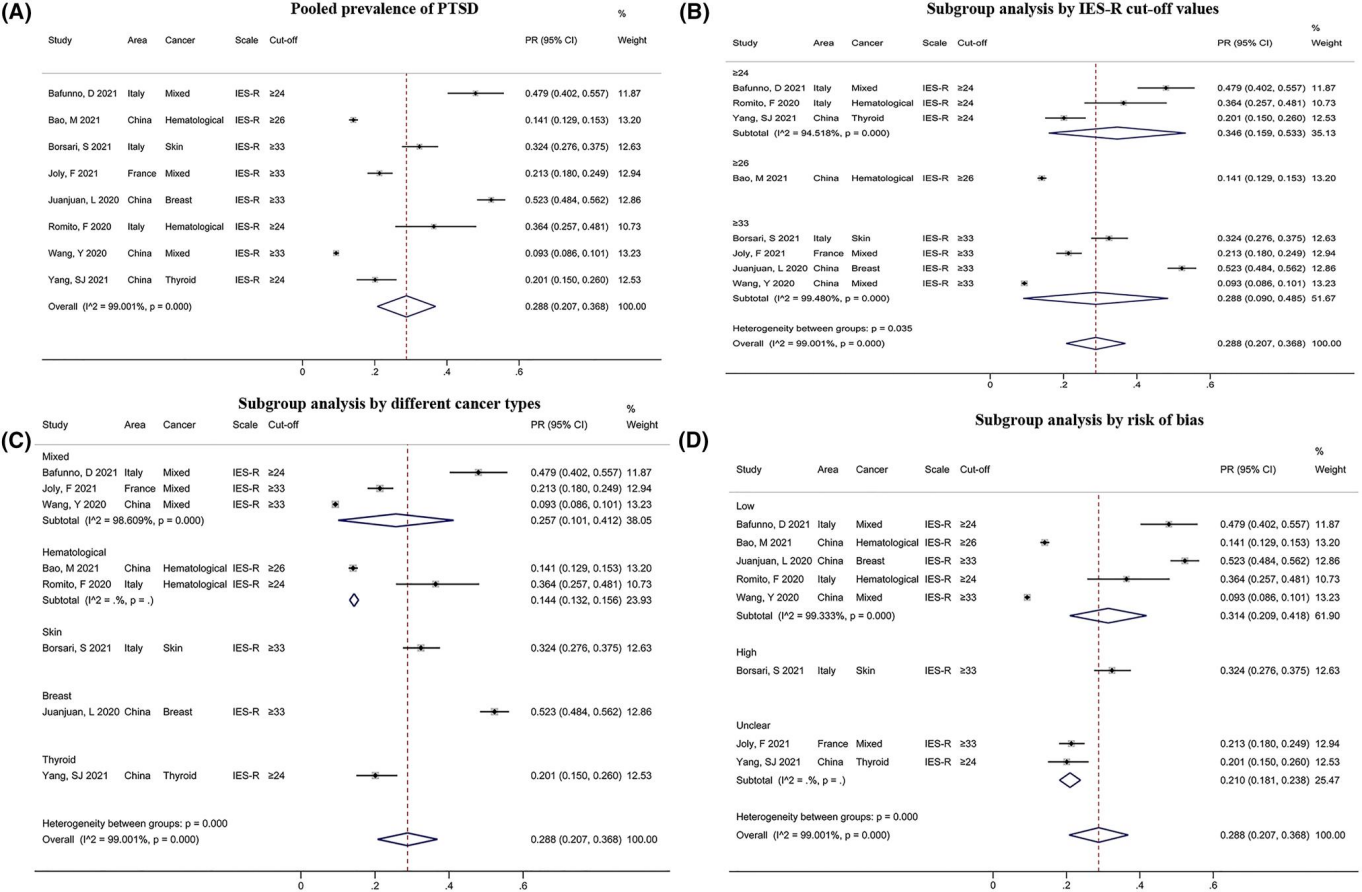
Forest plot of the PR of post‐traumatic stress disorder (PTSD) among patients with cancer. (A) Pooled PR of PTSD. (B) Subgroup analysis of the PR of PTSD based on IES‐R cut‐off values. (C) Subgroup analysis of the PR of PTSD based on different cancer types. (D) Subgroup analysis of the PR of PTSD based on risk of bias

The PR of PTSD was recorded in eight studies and all results were evaluated using the IES‐R scale. A significant heterogeneity existed (*I* ^*2*^ = 99.001%, *P* < 0.001), and the meta‐analysis showed the PR of PTSD among patients with cancer was 28.8% (95% CI: 0.207, 0.368, Figure 4A). A subgroup analysis using the IES‐R cut‐off values (≥24, ≥26, or≥ 33) was conducted. We found that the PR of PTSD was significantly higher among patients with cut‐off values ≥ 24 (34.6%, 95% CI: 0.159, 0.533) than those with values ≥ 26 (14.1%, 95% CI: 0.129, 0.153) or ≥ 33 (28.8%, 95% CI: 0.090, 0.486, Figure 4B). Significant results were observed in a subgroup analysis by cancer type (*P* < 0.01, Figure 4C) and risk of bias (unclear risk vs. high risk, *P* = 0.001, Figure 4D). Nevertheless, no significant results were found in the subgroup analysis by area (*P* > 0.05, Figure S3).

#### 3.3.4 Distress

**FIGURE 5:**
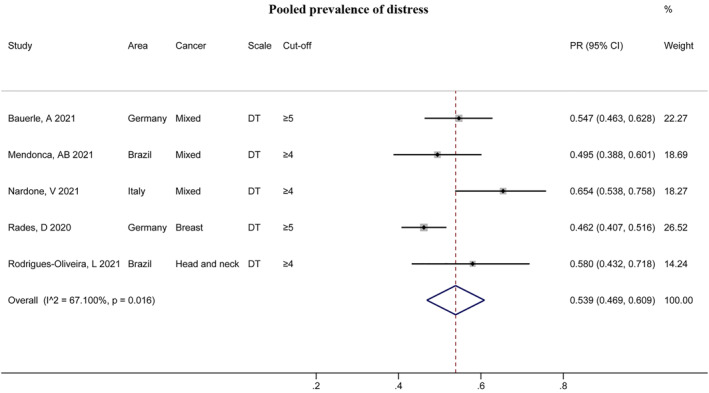
Forest plot of the PR of distress among patients with cancer

Five studies revealed the PR of distress (all assessed by DT scale), and the pooled result was 53.9% (95% CI: 0.469, 0.609, Figure 5) with significant heterogeneity (*I* ^2^ = 67.100%, *P* = 0.016). However, there were no significant differences in subgroup analyses classified by DT cutoff values, area, cancer types, and risk of bias (*P* > 0.05, Figure S4A‐4D).

#### 3.3.5 Insomnia

**FIGURE 6:**
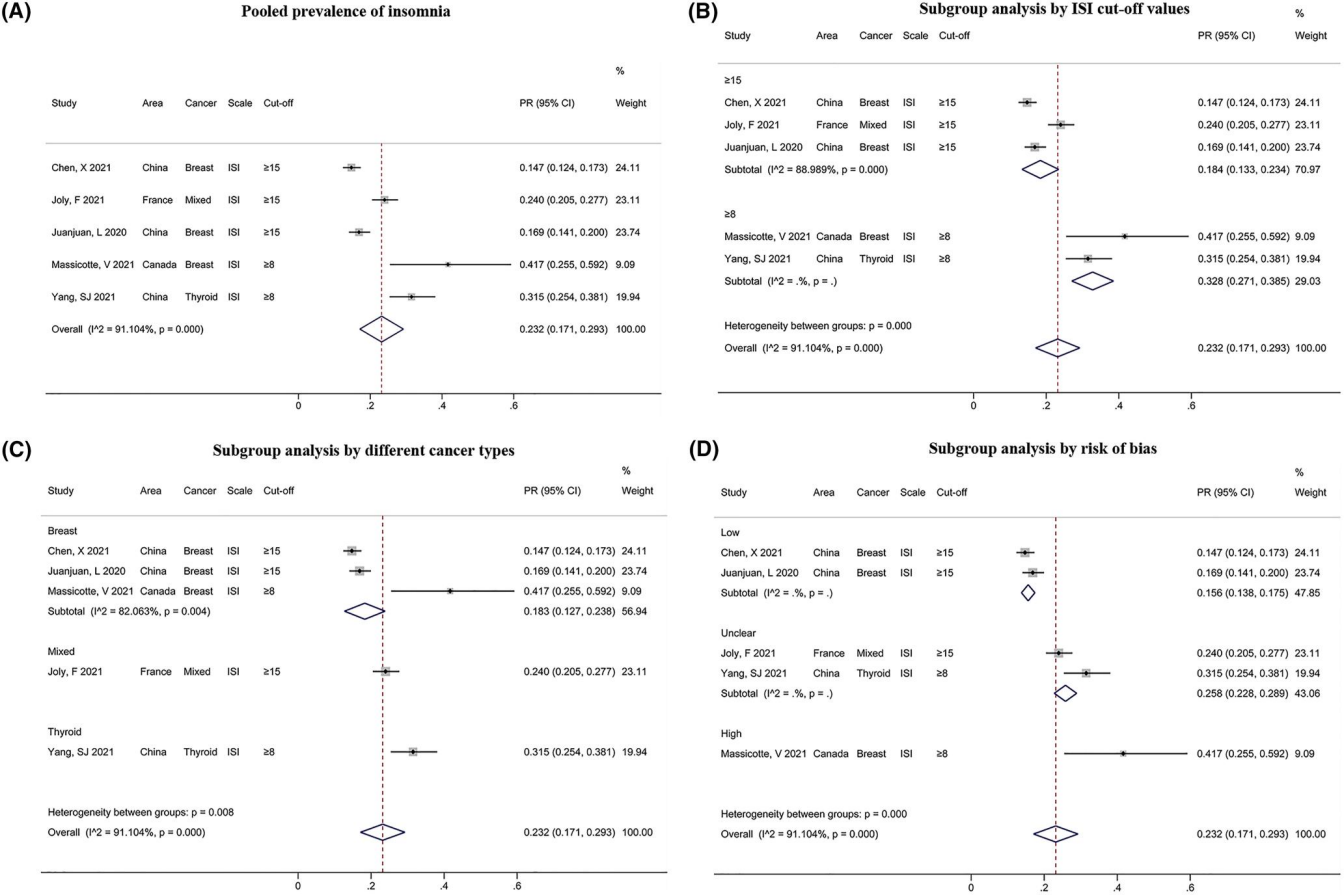
Forest plot of the PR of insomnia among patients with cancer. (A) Pooled PR of insomnia. (B) Subgroup analysis of the PR of insomnia based on ISI cut‐off values. (C) Subgroup analysis of the PR of insomnia based on different cancer types. (D) Subgroup analysis of the PR of insomnia based on risk of bias

Five studies used the ISI scale to assess PR in patients with insomnia. A meta‐analysis showed PR of insomnia among patients with cancer was 23.2% (95% CI: 0.171, 0.293, Figure 6A), with a significant heterogeneity of *I* ^*2*^ = 91.104% (*P* < 0.001). Subgroup analyses indicated that significant differences were found in ISI cutoff values, cancer types, and risk of bias (*P* < 0.01, Figures 6B–D). In brief, patients with ISI values ≥ 8 (32.8%, 95% CI: 0.271, 0.385) and thyroid cancer (31.5%, 95% CI: 0.254, 0.381) were at higher PR for insomnia. There was no difference in the subgroup analysis by area (Figure S5).

#### 3.3.6 Fear of cancer progression/recurrence

**FIGURE 7:**
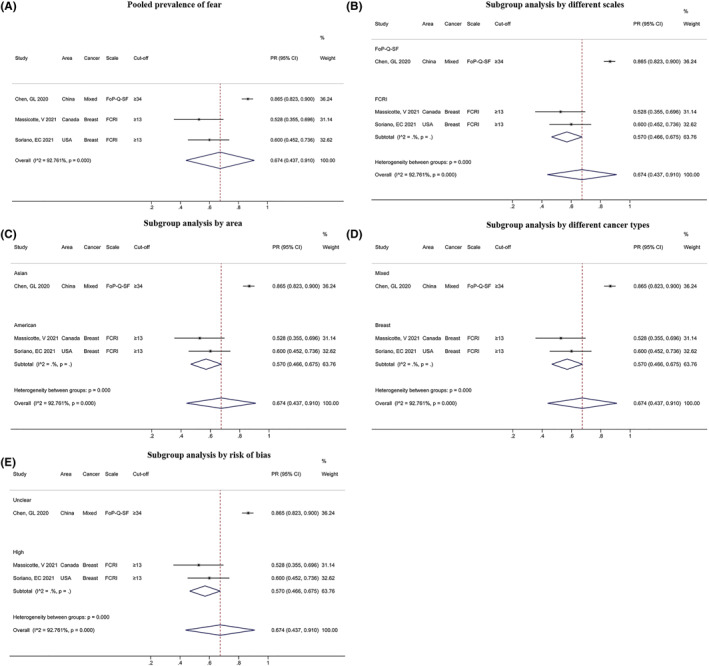
Forest plot of the PR of fear of cancer progression/recurrence among patients with cancer. (A) Pooled PR of fear. (B) Subgroup analysis of the PR of fear of cancer progression/recurrence based on different scales. (C) Subgroup analysis of the PR of fear of cancer progression/recurrence based on area. (D) Subgroup analysis of the PR of fear of cancer progression/recurrence based on different cancer types

Three studies reported the PR of fear of cancer progression/recurrence, and the pooled result was 67.4% (95% CI: 0.674, 0.910, Figure 7A) with significant heterogeneity (*I* ^*2*^ = 92.761%, *P* < 0.001). Moreover, significant results were observed in the subgroup analyses classified by differences in scale, area, cancer type, and risk of bias (*P* < 0.001, Figures 7B–E).

### 3.4 Stratified analysis

A stratified analysis was performed according to the PR of depression, anxiety, and PTSD. Four studies reported the stratified result of depression, and no statistical significance was found in fractionation by gender, marital status, and employment status (*P* > 0.05, Figure S6A‐S6C). Significant result was observed in education level, revealing that patients with education level of university or above (37.2%) had a higher PR for depression than those with high school or below (21.6%, *P* = 0.001, Figure S6D). Six studies recorded the stratified result of anxiety, and there was no statistical significance in fractionation by sex, marital status, employment status, and education level (*P* > 0.05, Figure S7). Regarding the prevalence of PTSD, three studies reported data on sex and employment status. A stratified analysis showed that female (27.9%) with cancer were significantly associated with higher PR of PTSD than male (17.9%, *P* < 0.01, Figure S8A); employed patients (47.4%) had an observably higher rate of PTSD than unemployed patients (37.7%, *P* < 0.01, Figure S8B).

### 3.5 Results of publication bias

**FIGURE 8:**
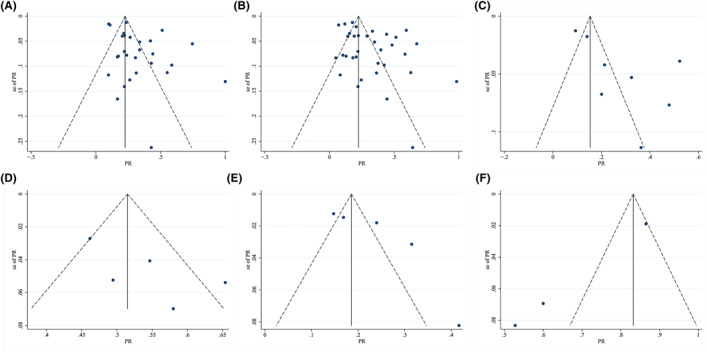
Funnel plots of publication bias for six mental health issues. (A) Depression. (B) Anxiety. (C), PTSD. (D) Distress. (E) Insomnia. (F) Fear of cancer progression/recurrence

Funnel plots for the six psychological issues were generated (Figures 8A–F). Asymmetry was observed in funnel plots for depression (Figure 8A), anxiety (Figure 8B), PTSD (Figure 8C), and fear of cancer progression/recurrence (Figure 8F). Meanwhile, Egger's test showed that potential publication bias was observed in depression (*P* = 0.019), anxiety (*P* = 0.009), PTSD (*P* = 0.038), and fear (*P* = 0.001) but not in distress (*P* = 0.139) and insomnia (*P* = 0.072).

## 4 DISCUSSION

This meta‐analysis included 40 studies that analyzed the PR of six psychological disorders in patients with cancer during the COVID‐19 pandemic. The pooled results indicated that 32.5%, 31.3%, 28.2%, 53.9%, 23.2%, and 67.4% of patients with cancer were affected by depression, anxiety, PTSD, distress, insomnia, and fear of cancer progression/recurrence, respectively. A subgroup analysis showed that the PR of depression, anxiety, and fear of cancer progression/recurrence estimated by different measuring scales was inconsistent; patients with head and neck cancer had the highest PR for depression and anxiety. Moreover, a stratified analysis revealed that patients with higher educational levels were more prone to depression; employed patients or women with cancer might tend to experience higher levels of PTSD.

In this study, we observed a high level of psychological disorder among cancer patients during the pandemic. The majority of cancer patients suffer from fear of cancer progression/recurrence. During the early pandemic, the government recommended postponing non‐emergency cancer surgery and routine cancer screening, resulting in a higher proportion of patients delaying or missing health care services.^57^ It has been indicated that the COVID‐19 pandemic can exacerbate the fear of disease progression or recurrence in patients with cancer due to access restrictions on follow‐up and treatment, imposed isolation restrictions, and the possibility that the healthcare systems becomes overworked.^58^ The vast majority of patients concern about the impact of delays on treatment and long‐term health. According to recent studies, the fact that most patients reported high fear of recurrence was due to concerns about lack of access to medical services, which were completely limited during the COVID‐19 pandemic.^59^ The level of distress in cancer patients has also increased due to treatment delays.^60^ Moreover, changes in treatment have led to concern and fear of disease recurrence in cancer patients, as well as increased levels of depression and anxiety.^61^ Therefore, with the continued spread of COVID‐19, more attention should be paid to its potentially harmful effects on the mental health of this particular population. However, only 1.6% of cancer patients sought psychological counseling during COVID‐19.^51^ We recommend that oncology clinics provide the necessary and timely mental health screening for cancer patients; accordingly, policymakers should develop personalized psychological care plans for cancer patients.

In this analysis, we used different scales to assess the PR of depression and anxiety, and the results were statistically significant. This may be caused by the different item numbers and scale constructions of each scale. In terms of depression rating scales, hospital anxiety and depression scale‐depression (HADS‐D, seven items) were designed to assess the emotional aspects of depression and exclude mental disorders caused by illness itself^62^; the self‐rating depression scale (SDS, 20 items) is used to measure the severity of psychological and physical symptoms of depression^63^; the PHQ‐9 is a self‐report assessment tool, and PHQ–2 (two items) and PHQ‐8 (eight items) are simplified from items in it.^64^ A previous study indicated that HADS‐D significantly underestimated depression in prostate cancer patients compared to PHQ‐9, and SDS showed a similar trend.^65^ Moreover, HADS could be a better option for depression assessment than SDS in patients with lung cancer.^66^ The State‐Trait Anxiety Inventory (STAI) is a self‐report measure of the severity of anxiety symptoms; HADS screens for clinically significant symptoms of anxiety in patients with medical conditions.^67^ Taken together, these different scales may have an impact on the detection of depression or anxiety in patients with cancer, which is consistent with our findings.

The prevalence of depression and anxiety varied according to cancer type. A previous study showed that a higher rate of depression/anxiety symptoms was observed in head and neck cancer,^68^ which was also found in this meta‐analysis. Patients with head and neck cancer suffer from unique challenges because much of social function depends on the structural and functional integrity of the head and neck. Psychological distress was also particularly prevalent among patients with head and neck cancer, with nearly 35% experiencing symptoms of depression and anxiety.^69^ In addition, patients with head and neck cancer have higher medical expenses compared to other cancer, especially during the COVID‐19 epidemic, which also brings additional financial burdens on patients and is a potential risk for the deterioration of their mental health.^70^, ^71^ Thus, it is necessary to consider the impact of anxiety and depression on clinical outcomes in the treatment of head and neck cancer.

In this study, several sociodemographic factors, such as education level, gender, and employment status, were associated with mental health problems, especially anxiety and PTSD. During the COVID‐19 outbreak, citizens' sources of information were mainly obtained through the media due to quarantine. However, disinformation spread on social media platforms may affect individuals' mental health.^72^ Thus, to discern this information, the educational level of the patients is crucial. Yang et al.^22^ indicated that patients with higher education had better awareness of cancer, especially in the context of the COVID‐19 pandemic. In addition, they have a strong ability to identify and process uncertain information and do not panic blindly, thereby avoiding or reducing anxiety and depression. However, we obtained inconsistent results, which should be confirmed in future studies with larger sample sizes. In general, stressors affect women more than men at the population level. Compared to men, the prevalence of PTSD and anxiety was increased in women among cancer and non‐cancer patients.^73^ We also observed that women were more vulnerable to PTSD during this pandemic. COVID‐19 was with a major impact on the global economy and individual employment. As a result of the COVID‐19 outbreak and social lockdown policy, approximately eight million jobs were furloughed or unplanned loss of employment in the UK. The impact of COVID‐19 on change in employment status has left women with breast cancer vulnerable to affective disorders and poor cognitive function.^74^ Forced unemployment and income problems caused by the pandemic are associated with greater psychological distress. For example, working‐age patients with hematology diseases may have lost their job during the COVID‐19 pandemic, or may have to weigh the benefits of work against the potential increased risk of contracting COVID‐19, and thus the reduced income may lead to interruptions in cancer treatment and more severe psychological problems, such as depression and anxiety.^75^ Therefore, the decline in economic levels had a greater impact on employed patients than on the unemployed. This phenomenon was also observed in this meta‐analysis.

## 5 CLINICAL IMPLICATION

This meta‐analysis has several advantages and practical implications. First, a large number of studies with large sample sizes were included in the analysis. Second, a merged meta‐analysis revealed that cancer patients had varying degrees of mental health problems. Oncologists may ignore the psychological problems of patients when formulating cancer treatment plans. Thus, our meta‐analysis suggests that a comprehensive assessment of the prevalence of psychological disorder is necessary before providing optimal care to cancer patients in clinical practice.^76^ We call for the need to develop psychological interventions for cancer patients to improve their quality of life and reduce their levels of mental problems.^77^ Third, the methodological quality of the included studies was high, and the results of the meta‐analysis were reliable.

## 6 STUDY LIMITATIONS

This meta‐analysis has some limitations. First, a significant heterogeneity was observed among the included studies. This may be caused by the type of cancer and different measuring scales or cut‐off values. Second, there may be interactive effects among some clinical factors such as cancer stage, education level, and work status. Nevertheless, due to limited statistical methods, it is difficult to explore the source of heterogeneity and the impact of these factors on the results through quantitative analysis. Third, significant publication bias was observed in some variables (e.g., depression and anxiety), which may underestimate the prevalence of mental health issues. In future meta‐analyses, stricter criteria, such as a limited evaluation scale and cancer type, should be adopted when selecting the included articles.

## 7 CONCLUSION

Our meta‐analysis revealed the PR of depression, anxiety, PTSD, distress, insomnia, and fear of cancer progression/recurrence among cancer patients during the COVID‐19 pandemic by integrating existing evidence. These findings support that the mental health of individuals with cancer should receive more attention under pandemic conditions.

## AUTHOR CONTRIBUTIONS

Lemeng Zhang, Xiaohong Liu, Fei Tong carried out the conception and design of the research. Ran Zou, Wanglian Peng, Hui Yang, Feng Liu and Desong Yang participated in the acquisition of data. Xufen Huang, Minni Wen, Ling Jiang and Lili Yi carried out the analysis and interpretation of data. Lemeng Zhang, Xiaohong Liu participated in the design of the study, prepare and revise the manuscript. All authors read and approved the final manuscript.

## CONFLICT OF INTEREST

The authors have declared that no conflict of interest exists.

## Supporting information

Figure S1

Figure S2

Figure S3

Figure S4

Figure S5

Figure S6

Figure S7

Figure S8

Supporting Information S1

## Data Availability

Data sharing is not applicable to this article as no new data were created or analyzed in this study.
