# Supplementary figures and images for: The prevalence of psychological disorders among cancer patients during the COVID‐19 pandemic: A meta‐analysis
Source: Psychooncology. 2022 Aug 19:10.1002/pon.6012. Online ahead of print. doi: 10.1002/pon.6012 (PMC9538248; doi:10.1002/pon.6012)

A

## Subgroup analysis by area

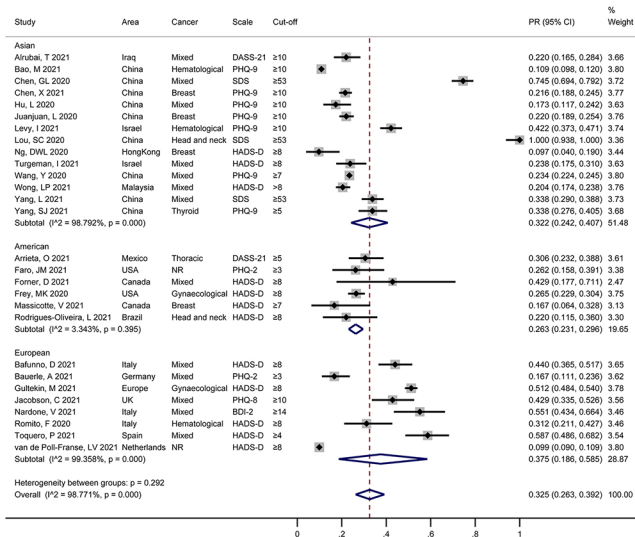

B

## Subgroup analysis by risk of bias

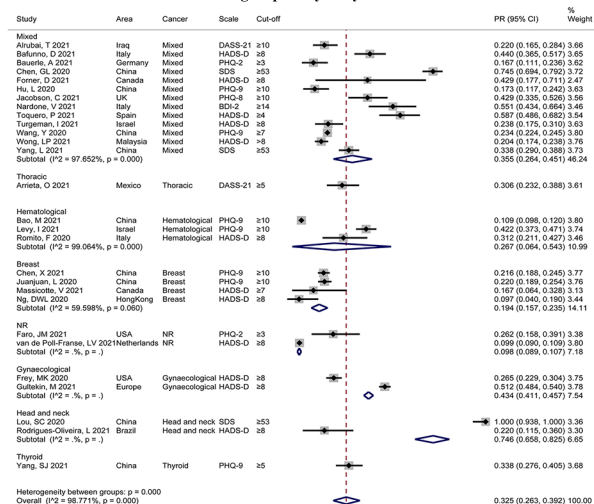

Supplement: Supplementary file 1 — Figure S1 [file PON-9999-0-s009.pdf]

A

Subgroup analysis by area

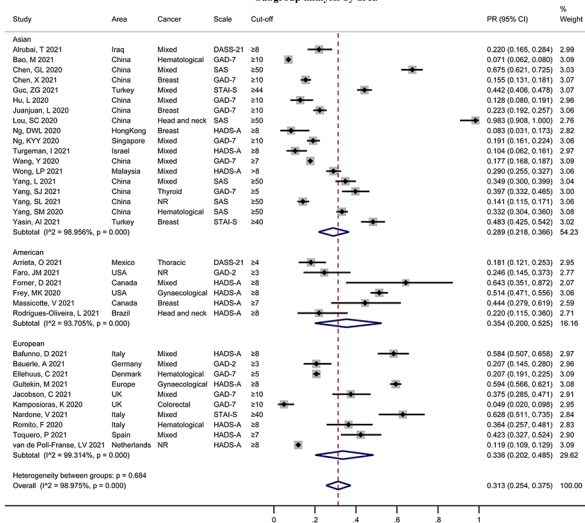

B

Subgroup analysis by risk of bias

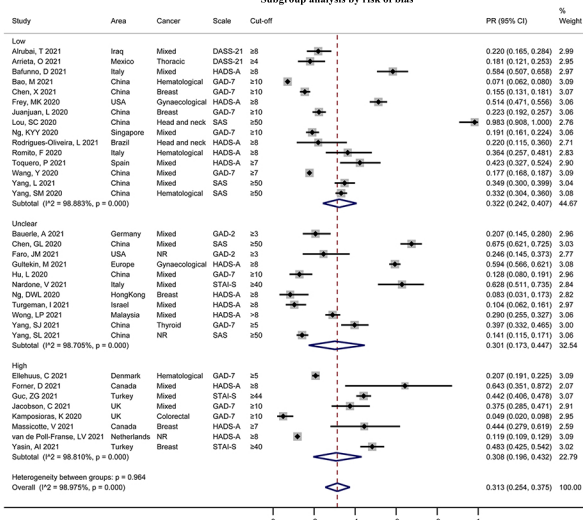

Supplement: Supplementary file 2 — Figure S2 [file PON-9999-0-s008.pdf]

# Subgroup analysis by area

%

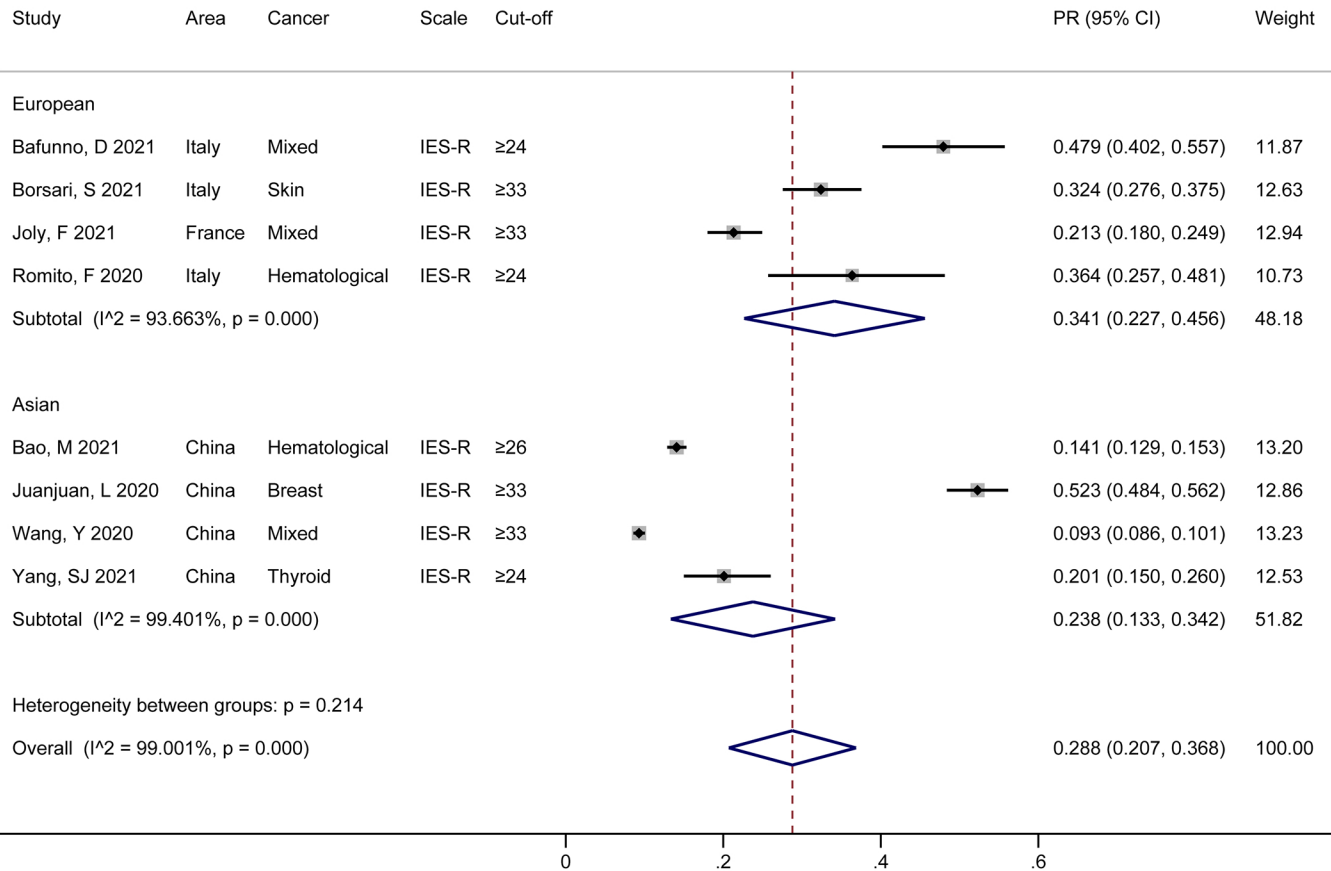

Supplement: Supplementary file 3 — Figure S3 [file PON-9999-0-s004.pdf]

Subgroup analysis by area

%

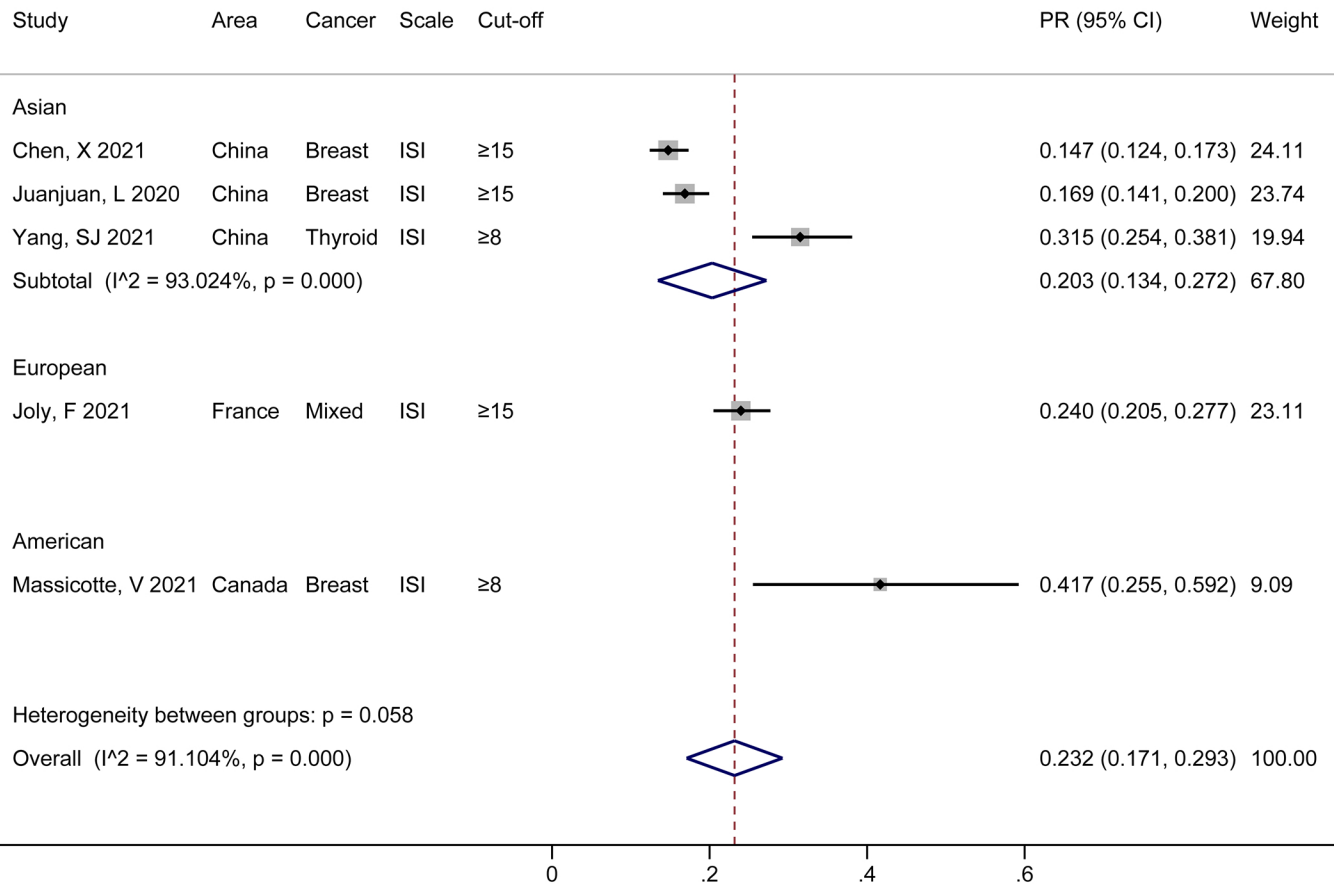

Supplement: Supplementary file 5 — Figure S5 [file PON-9999-0-s002.pdf]

A

## Stratified analysis by gender

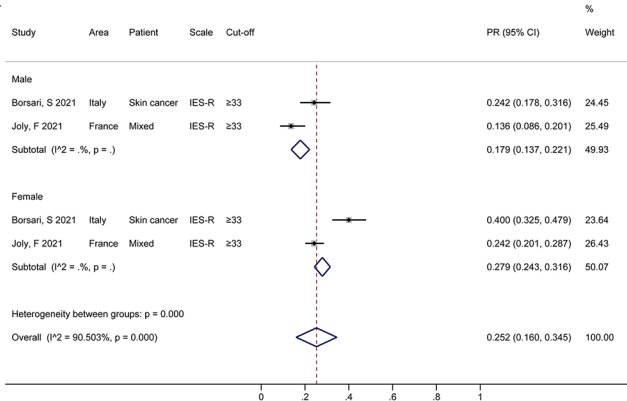

B

## Stratified analysis by employment status

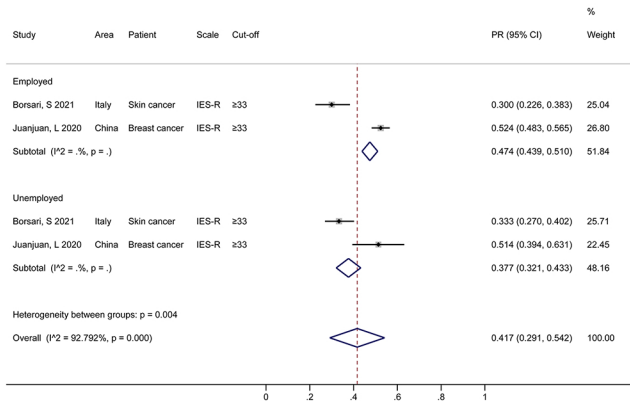

Supplement: Supplementary file 8 — Figure S8 [file PON-9999-0-s005.pdf]
