# Supplementary material for: The prevalence of psychological disorders among cancer patients during the COVID‐19 pandemic: A meta‐analysis
Source: Psychooncology. 2022 Aug 19:10.1002/pon.6012. Online ahead of print. doi: 10.1002/pon.6012 (PMC9538248; doi:10.1002/pon.6012)
Supplement: Supplementary file 4 — Figure S4 [file PON-9999-0-s007.pdf]

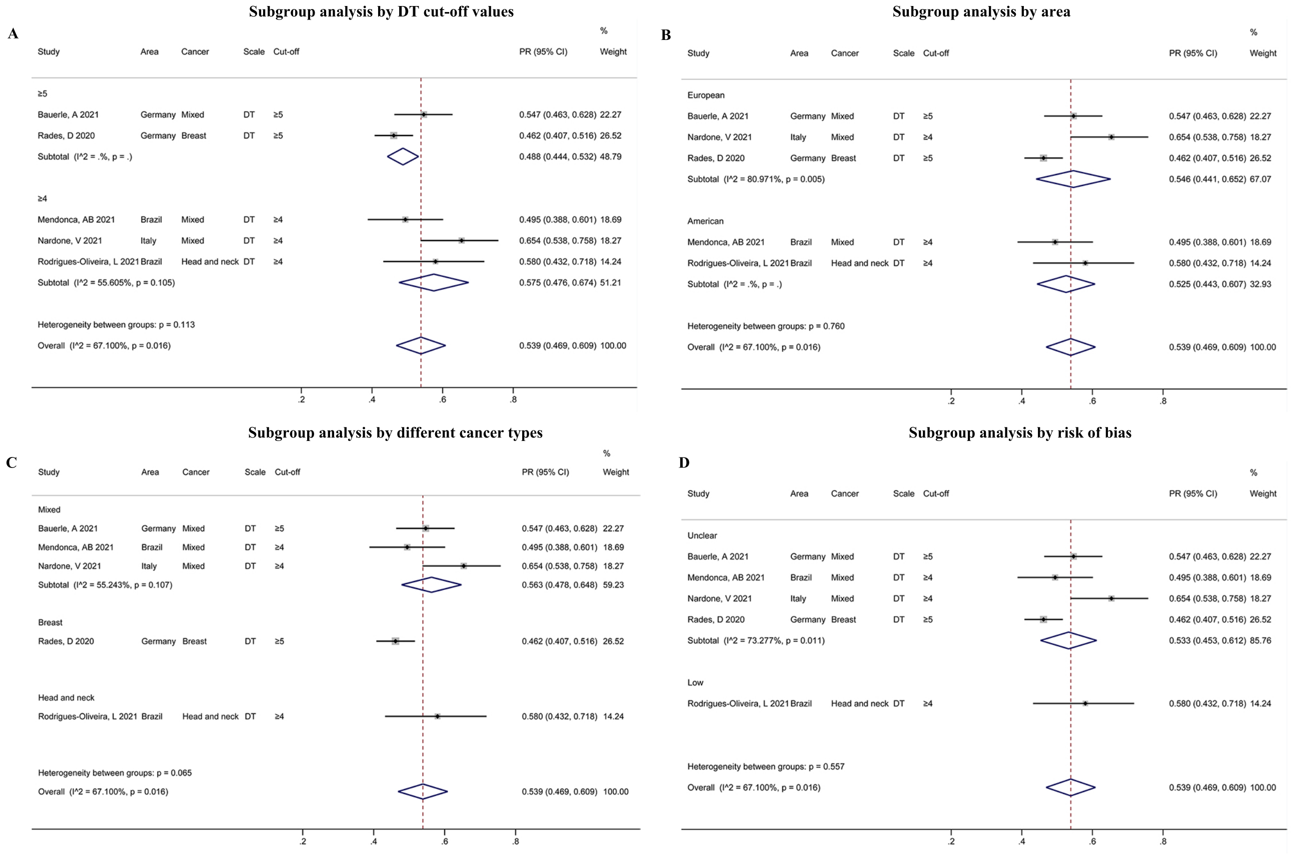
Subgroup analysis by DT cut-off values
A
Study Area Cancer Scale Cut-off PR (95% CI) % Weight
≥5
Bauerle, A 2021 Germany Mixed DT ≥5 0.547 (0.463, 0.628) 22.27
Rades, D 2020 Germany Breast DT ≥5 0.462 (0.407, 0.516) 26.52
Subtotal (I^2 = .%, p = .) 0.488 (0.444, 0.532) 48.79
≥4
Mendonca, AB 2021 Brazil Mixed DT ≥4 0.495 (0.388, 0.601) 18.69
Nardone, V 2021 Italy Mixed DT ≥4 0.654 (0.538, 0.758) 18.27
Rodrigues-Oliveira, L 2021 Brazil Head and neck DT ≥4 0.580 (0.432, 0.718) 14.24
Subtotal (I^2 = 55.605%, p = 0.105) 0.575 (0.476, 0.674) 51.21
Heterogeneity between groups: p = 0.113
Overall (I^2 = 67.100%, p = 0.016) 0.539 (0.469, 0.609) 100.00
.2 .4 .6 .8
Subgroup analysis by area
B
Study Area Cancer Scale Cut-off PR (95% CI) % Weight
European
Bauerle, A 2021 Germany Mixed DT ≥5 0.547 (0.463, 0.628) 22.27
Nardone, V 2021 Italy Mixed DT ≥4 0.654 (0.538, 0.758) 18.27
Rades, D 2020 Germany Breast DT ≥5 0.462 (0.407, 0.516) 26.52
Subtotal (I^2 = 80.971%, p = 0.005) 0.546 (0.441, 0.652) 67.07
American
Mendonca, AB 2021 Brazil Mixed DT ≥4 0.495 (0.388, 0.601) 18.69
Rodrigues-Oliveira, L 2021 Brazil Head and neck DT ≥4 0.580 (0.432, 0.718) 14.24
Subtotal (I^2 = .%, p = .) 0.525 (0.443, 0.607) 32.93
Heterogeneity between groups: p = 0.760
Overall (I^2 = 67.100%, p = 0.016) 0.539 (0.469, 0.609) 100.00
.2 .4 .6 .8
Subgroup analysis by different cancer types
C
Study Area Cancer Scale Cut-off PR (95% CI) % Weight
Mixed
Bauerle, A 2021 Germany Mixed DT ≥5 0.547 (0.463, 0.628) 22.27
Mendonca, AB 2021 Brazil Mixed DT ≥4 0.495 (0.388, 0.601) 18.69
Nardone, V 2021 Italy Mixed DT ≥4 0.654 (0.538, 0.758) 18.27
Subtotal (I^2 = 55.243%, p = 0.107) 0.563 (0.478, 0.648) 59.23
Breast
Rades, D 2020 Germany Breast DT ≥5 0.462 (0.407, 0.516) 26.52
Head and neck
Rodrigues-Oliveira, L 2021 Brazil Head and neck DT ≥4 0.580 (0.432, 0.718) 14.24
Heterogeneity between groups: p = 0.065
Overall (I^2 = 67.100%, p = 0.016) 0.539 (0.469, 0.609) 100.00
.2 .4 .6 .8
Subgroup analysis by risk of bias
D
Study Area Cancer Scale Cut-off PR (95% CI) % Weight
Unclear
Bauerle, A 2021 Germany Mixed DT ≥5 0.547 (0.463, 0.628) 22.27
Mendonca, AB 2021 Brazil Mixed DT ≥4 0.495 (0.388, 0.601) 18.69
Nardone, V 2021 Italy Mixed DT ≥4 0.654 (0.538, 0.758) 18.27
Rades, D 2020 Germany Breast DT ≥5 0.462 (0.407, 0.516) 26.52
Subtotal (I^2 = 73.277%, p = 0.011) 0.533 (0.453, 0.612) 85.76
Low
Rodrigues-Oliveira, L 2021 Brazil Head and neck DT ≥4 0.580 (0.432, 0.718) 14.24
Heterogeneity between groups: p = 0.557
Overall (I^2 = 67.100%, p = 0.016) 0.539 (0.469, 0.609) 100.00
.2 .4 .6 .8
