# Supplementary material for: The prevalence of psychological disorders among cancer patients during the COVID‐19 pandemic: A meta‐analysis
Source: Psychooncology. 2022 Aug 19:10.1002/pon.6012. Online ahead of print. doi: 10.1002/pon.6012 (PMC9538248; doi:10.1002/pon.6012)
Supplement: Supplementary file 6 — Figure S6 [file PON-9999-0-s003.pdf]

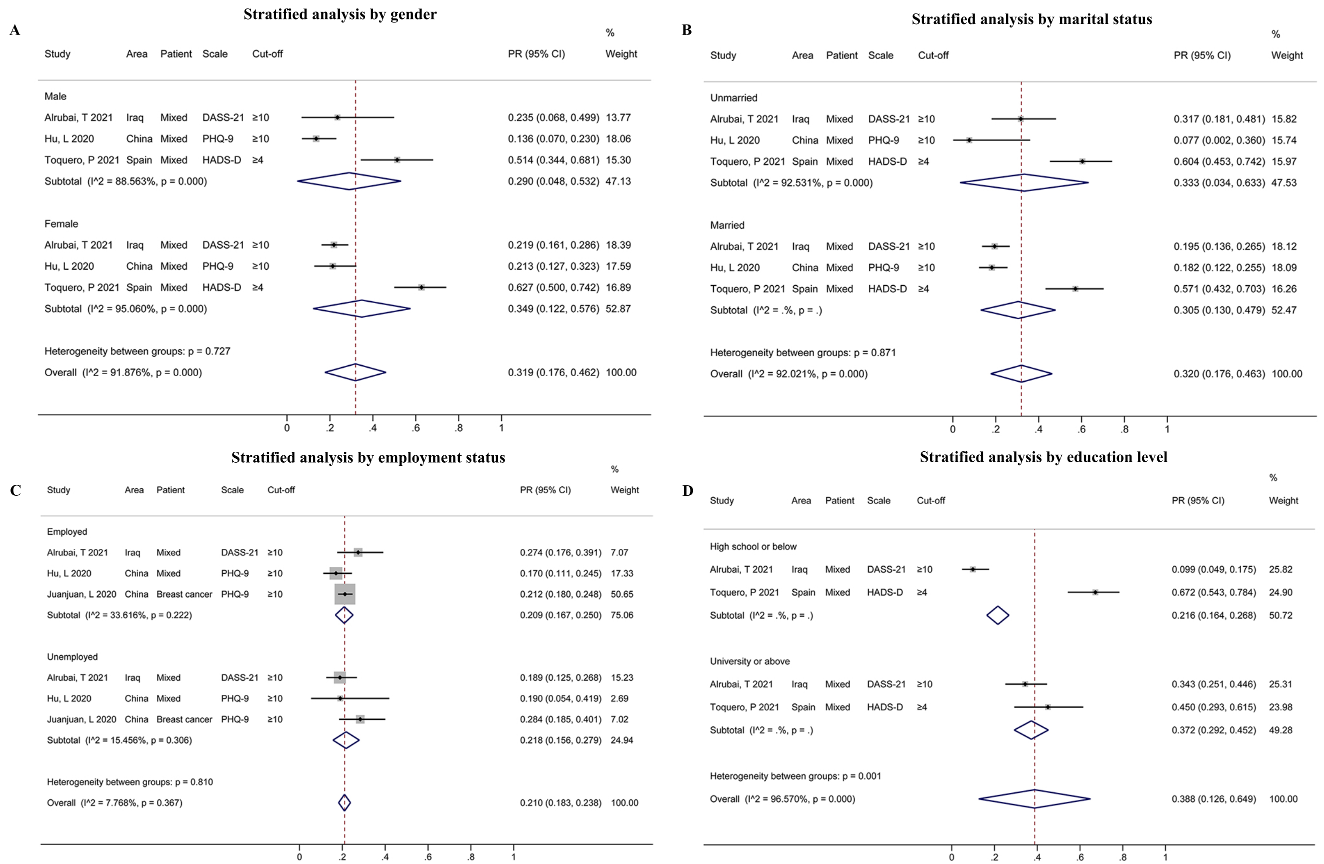
A
Stratified analysis by gender
Study Area Patient Scale Cut-off PR (95% CI) % Weight
Male
Alrubai, T 2021 Iraq Mixed DASS-21 ≥10 0.235 (0.068, 0.499) 13.77
Hu, L 2020 China Mixed PHQ-9 ≥10 0.136 (0.070, 0.230) 18.06
Toquero, P 2021 Spain Mixed HADS-D ≥4 0.514 (0.344, 0.681) 15.30
Subtotal (I^2 = 88.563%, p = 0.000) 0.290 (0.048, 0.532) 47.13
Female
Alrubai, T 2021 Iraq Mixed DASS-21 ≥10 0.219 (0.161, 0.286) 18.39
Hu, L 2020 China Mixed PHQ-9 ≥10 0.213 (0.127, 0.323) 17.59
Toquero, P 2021 Spain Mixed HADS-D ≥4 0.627 (0.500, 0.742) 16.89
Subtotal (I^2 = 95.060%, p = 0.000) 0.349 (0.122, 0.576) 52.87
Heterogeneity between groups: p = 0.727
Overall (I^2 = 91.876%, p = 0.000) 0.319 (0.176, 0.462) 100.00
0 .2 .4 .6 .8 1
B
Stratified analysis by marital status
Study Area Patient Scale Cut-off PR (95% CI) % Weight
Unmarried
Alrubai, T 2021 Iraq Mixed DASS-21 ≥10 0.317 (0.181, 0.481) 15.82
Hu, L 2020 China Mixed PHQ-9 ≥10 0.077 (0.002, 0.360) 15.74
Toquero, P 2021 Spain Mixed HADS-D ≥4 0.604 (0.453, 0.742) 15.97
Subtotal (I^2 = 92.531%, p = 0.000) 0.333 (0.034, 0.633) 47.53
Married
Alrubai, T 2021 Iraq Mixed DASS-21 ≥10 0.195 (0.136, 0.265) 18.12
Hu, L 2020 China Mixed PHQ-9 ≥10 0.182 (0.122, 0.255) 18.09
Toquero, P 2021 Spain Mixed HADS-D ≥4 0.571 (0.432, 0.703) 16.26
Subtotal (I^2 = .%, p = .) 0.305 (0.130, 0.479) 52.47
Heterogeneity between groups: p = 0.871
Overall (I^2 = 92.021%, p = 0.000) 0.320 (0.176, 0.463) 100.00
0 .2 .4 .6 .8 1
C
Stratified analysis by employment status
Study Area Patient Scale Cut-off PR (95% CI) % Weight
Employed
Alrubai, T 2021 Iraq Mixed DASS-21 ≥10 0.274 (0.176, 0.391) 7.07
Hu, L 2020 China Mixed PHQ-9 ≥10 0.170 (0.111, 0.245) 17.33
Juanjuan, L 2020 China Breast cancer PHQ-9 ≥10 0.212 (0.180, 0.248) 50.65
Subtotal (I^2 = 33.616%, p = 0.222) 0.209 (0.167, 0.250) 75.06
Unemployed
Alrubai, T 2021 Iraq Mixed DASS-21 ≥10 0.189 (0.125, 0.268) 15.23
Hu, L 2020 China Mixed PHQ-9 ≥10 0.190 (0.054, 0.419) 2.69
Juanjuan, L 2020 China Breast cancer PHQ-9 ≥10 0.284 (0.185, 0.401) 7.02
Subtotal (I^2 = 15.456%, p = 0.306) 0.218 (0.156, 0.279) 24.94
Heterogeneity between groups: p = 0.810
Overall (I^2 = 7.768%, p = 0.367) 0.210 (0.183, 0.238) 100.00
0 .2 .4 .6 .8 1
D
Stratified analysis by education level
Study Area Patient Scale Cut-off PR (95% CI) % Weight
High school or below
Alrubai, T 2021 Iraq Mixed DASS-21 ≥10 0.099 (0.049, 0.175) 25.82
Toquero, P 2021 Spain Mixed HADS-D ≥4 0.672 (0.543, 0.784) 24.90
Subtotal (I^2 = .%, p = .) 0.216 (0.164, 0.268) 50.72
University or above
Alrubai, T 2021 Iraq Mixed DASS-21 ≥10 0.343 (0.251, 0.446) 25.31
Toquero, P 2021 Spain Mixed HADS-D ≥4 0.450 (0.293, 0.615) 23.98
Subtotal (I^2 = .%, p = .) 0.372 (0.292, 0.452) 49.28
Heterogeneity between groups: p = 0.001
Overall (I^2 = 96.570%, p = 0.000) 0.388 (0.126, 0.649) 100.00
0 .2 .4 .6 .8 1
