# Supplementary material for: The prevalence of psychological disorders among cancer patients during the COVID‐19 pandemic: A meta‐analysis
Source: Psychooncology. 2022 Aug 19:10.1002/pon.6012. Online ahead of print. doi: 10.1002/pon.6012 (PMC9538248; doi:10.1002/pon.6012)
Supplement: Supplementary file 7 — Figure S7 [file PON-9999-0-s006.pdf]

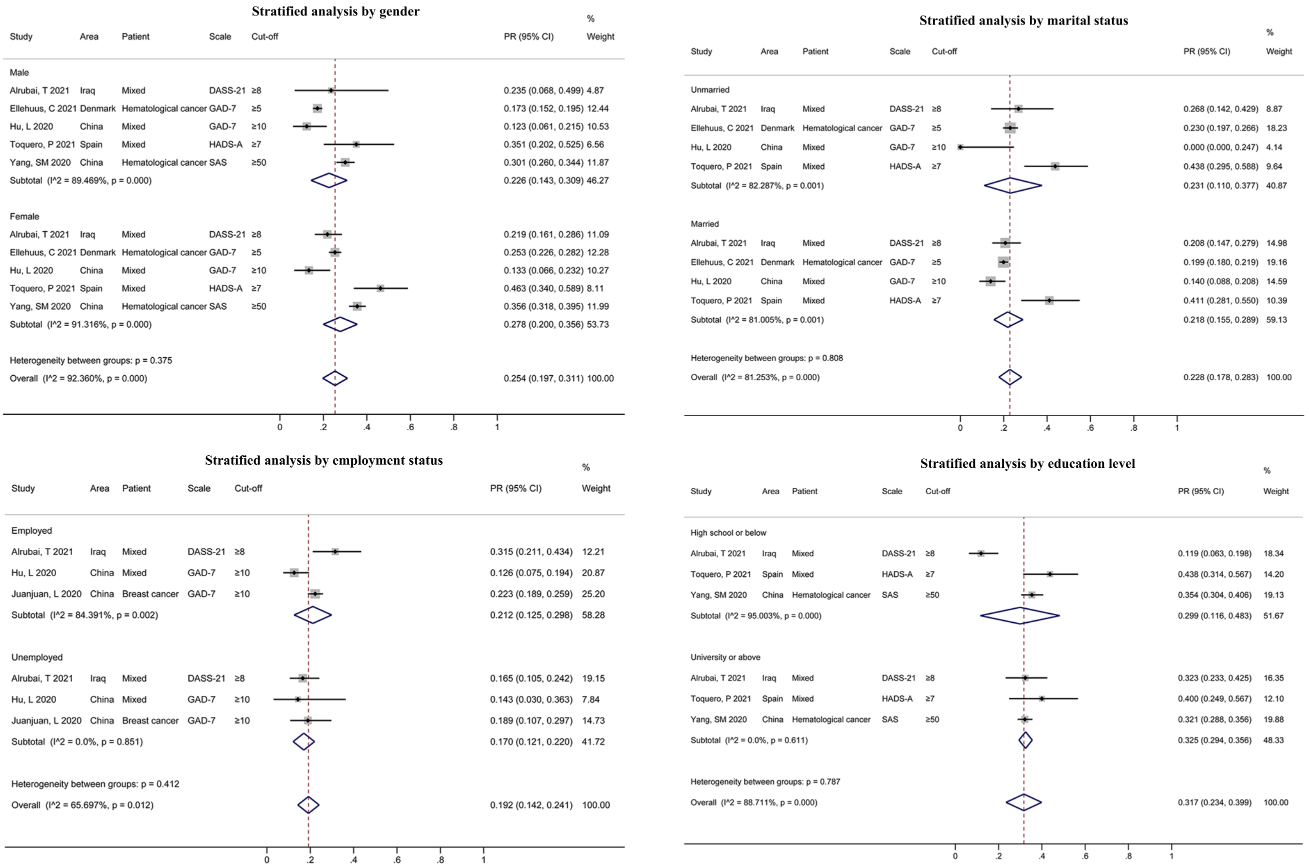

Stratified analysis by gender
Study Area Patient Scale Cut-off PR (95% CI) % Weight
Male
Alrubai, T 2021 Iraq Mixed DASS-21 ≥8 0.235 (0.068, 0.499) 4.87
Ellehuus, C 2021 Denmark Hematological cancer GAD-7 ≥5 0.173 (0.152, 0.195) 12.44
Hu, L 2020 China Mixed GAD-7 ≥10 0.123 (0.061, 0.215) 10.53
Toquero, P 2021 Spain Mixed HADS-A ≥7 0.351 (0.202, 0.525) 6.56
Yang, SM 2020 China Hematological cancer SAS ≥50 0.301 (0.260, 0.344) 11.87
Subtotal (I^2 = 89.469%, p = 0.000) 0.226 (0.143, 0.309) 46.27
Female
Alrubai, T 2021 Iraq Mixed DASS-21 ≥8 0.219 (0.161, 0.286) 11.09
Ellehuus, C 2021 Denmark Hematological cancer GAD-7 ≥5 0.253 (0.226, 0.282) 12.28
Hu, L 2020 China Mixed GAD-7 ≥10 0.133 (0.066, 0.232) 10.27
Toquero, P 2021 Spain Mixed HADS-A ≥7 0.463 (0.340, 0.589) 8.11
Yang, SM 2020 China Hematological cancer SAS ≥50 0.356 (0.318, 0.395) 11.99
Subtotal (I^2 = 91.316%, p = 0.000) 0.278 (0.200, 0.356) 53.73
Heterogeneity between groups: p = 0.375
Overall (I^2 = 92.360%, p = 0.000) 0.254 (0.197, 0.311) 100.00
0 .2 .4 .6 .8 1
Stratified analysis by marital status
Study Area Patient Scale Cut-off PR (95% CI) % Weight
Unmarried
Alrubai, T 2021 Iraq Mixed DASS-21 ≥8 0.268 (0.142, 0.429) 8.87
Ellehuus, C 2021 Denmark Hematological cancer GAD-7 ≥5 0.230 (0.197, 0.266) 18.23
Hu, L 2020 China Mixed GAD-7 ≥10 0.000 (0.000, 0.247) 4.14
Toquero, P 2021 Spain Mixed HADS-A ≥7 0.438 (0.295, 0.588) 9.64
Subtotal (I^2 = 82.287%, p = 0.001) 0.231 (0.110, 0.377) 40.87
Married
Alrubai, T 2021 Iraq Mixed DASS-21 ≥8 0.208 (0.147, 0.279) 14.98
Ellehuus, C 2021 Denmark Hematological cancer GAD-7 ≥5 0.199 (0.180, 0.219) 19.16
Hu, L 2020 China Mixed GAD-7 ≥10 0.140 (0.088, 0.208) 14.59
Toquero, P 2021 Spain Mixed HADS-A ≥7 0.411 (0.281, 0.550) 10.39
Subtotal (I^2 = 81.005%, p = 0.001) 0.218 (0.155, 0.289) 59.13
Heterogeneity between groups: p = 0.808
Overall (I^2 = 81.253%, p = 0.000) 0.228 (0.178, 0.283) 100.00
0 .2 .4 .6 .8 1
Stratified analysis by employment status
Study Area Patient Scale Cut-off PR (95% CI) % Weight
Employed
Alrubai, T 2021 Iraq Mixed DASS-21 ≥8 0.315 (0.211, 0.434) 12.21
Hu, L 2020 China Mixed GAD-7 ≥10 0.126 (0.075, 0.194) 20.87
Juanjuan, L 2020 China Breast cancer GAD-7 ≥10 0.223 (0.189, 0.259) 25.20
Subtotal (I^2 = 84.391%, p = 0.002) 0.212 (0.125, 0.298) 58.28
Unemployed
Alrubai, T 2021 Iraq Mixed DASS-21 ≥8 0.165 (0.105, 0.242) 19.15
Hu, L 2020 China Mixed GAD-7 ≥10 0.143 (0.030, 0.363) 7.84
Juanjuan, L 2020 China Breast cancer GAD-7 ≥10 0.189 (0.107, 0.297) 14.73
Subtotal (I^2 = 0.0%, p = 0.851) 0.170 (0.121, 0.220) 41.72
Heterogeneity between groups: p = 0.412
Overall (I^2 = 65.697%, p = 0.012) 0.192 (0.142, 0.241) 100.00
0 .2 .4 .6 .8 1
Stratified analysis by education level
Study Area Patient Scale Cut-off PR (95% CI) % Weight
High school or below
Alrubai, T 2021 Iraq Mixed DASS-21 ≥8 0.119 (0.063, 0.198) 18.34
Toquero, P 2021 Spain Mixed HADS-A ≥7 0.438 (0.314, 0.567) 14.20
Yang, SM 2020 China Hematological cancer SAS ≥50 0.354 (0.304, 0.406) 19.13
Subtotal (I^2 = 95.003%, p = 0.000) 0.299 (0.116, 0.483) 51.67
University or above
Alrubai, T 2021 Iraq Mixed DASS-21 ≥8 0.323 (0.233, 0.425) 16.35
Toquero, P 2021 Spain Mixed HADS-A ≥7 0.400 (0.249, 0.567) 12.10
Yang, SM 2020 China Hematological cancer SAS ≥50 0.321 (0.288, 0.356) 19.88
Subtotal (I^2 = 0.0%, p = 0.611) 0.325 (0.294, 0.356) 48.33
Heterogeneity between groups: p = 0.787
Overall (I^2 = 88.711%, p = 0.000) 0.317 (0.234, 0.399) 100.00
0 .2 .4 .6 .8 1
