# Supplementary material for: The prevalence of psychological disorders among cancer patients during the COVID‐19 pandemic: A meta‐analysis
Source: Psychooncology. 2022 Aug 19:10.1002/pon.6012. Online ahead of print. doi: 10.1002/pon.6012 (PMC9538248; doi:10.1002/pon.6012)
Supplement: Supplementary file 9 — Supporting Information S1 [file PON-9999-0-s001.docx]

**Table S1. Selection strategy in PubMed** (The retrieval time: 20220110)

| Search | Query | Items found |
| --- | --- | --- |
| #1 | "neoplasms"[MeSH Terms] OR "neoplasms"[tiab] OR "neoplasm"[tiab] OR "cancerated"[tiab] OR "canceration"[tiab] OR "cancerization"[tiab] OR "cancerized"[tiab] OR "cancerous"[tiab] OR "cancer"[tiab] OR "cancers"[tiab] OR "cysts"[MeSH Terms] OR "cysts"[tiab] OR "cyst"[tiab] OR "neurofibroma"[MeSH Terms] OR "neurofibroma"[tiab] OR "neurofibromas"[tiab] OR "tumoral"[tiab] OR "tumorous"[tiab] OR "tumour"[tiab] OR "tumor"[tiab] OR "tumoural"[tiab] OR "tumourous"[tiab] OR "tumours"[tiab] OR "tumors"[tiab] | 4586595 |
| #2 | "covid 19"[tiab] OR "covid 19"[MeSH Terms] OR "covid 19 vaccines"[tiab] OR "covid 19 vaccines"[MeSH Terms] OR "covid 19 serotherapy"[tiab] OR "covid 19 serotherapy"[Supplementary Concept] OR "covid 19 nucleic acid testing"[tiab] OR "covid 19 nucleic acid testing"[MeSH Terms] OR "covid 19 serological testing"[tiab] OR "covid 19 serological testing"[MeSH Terms] OR "covid 19 testing"[tiab] OR "covid 19 testing"[MeSH Terms] OR "sars cov 2"[tiab] OR "sars cov 2"[MeSH Terms] OR "severe acute respiratory syndrome coronavirus 2"[tiab] OR "ncov"[tiab] OR "2019 ncov"[tiab] OR ("sars cov 2"[MeSH Terms] OR "sars cov 2"[tiab] OR "sars cov 2"[tiab]) | 209080 |
| #3 | ("depressed"[tiab] OR "depression"[MeSH Terms] OR "depression"[tiab] OR "depressions"[tiab] OR "depressive disorder"[MeSH Terms] OR ("depressive"[tiab] AND "disorder"[tiab]) OR "depressive disorder"[tiab] OR "depressivity"[tiab] OR "depressive"[tiab] OR "depressively"[tiab] OR "depressiveness"[tiab] OR "depressives"[tiab]) OR ("distress"[tiab] OR "distressed"[tiab] OR "distresses"[tiab] OR "distressful"[tiab] OR "distressing"[tiab]) OR ("stress"[tiab] OR "stressed"[tiab] OR "stresses"[tiab] OR "stressful"[tiab] OR "stressfulness"[tiab] OR "stressing"[tiab]) OR ("anxiety"[MeSH Terms] OR "anxiety"[tiab] OR "anxieties"[tiab]) OR ("post-traumatic stress symptoms"[tiab] OR "posttraumatic stress disorder"[tiab] OR ("stress disorders, post traumatic"[MeSH Terms] OR ("stress"[tiab] AND "disorders"[tiab] AND "post traumatic"[tiab]) OR "post-traumatic stress disorders"[tiab] OR "ptsd"[tiab])) OR ("burnout, psychological"[MeSH Terms] OR ("burnout"[tiab] AND "psychological"[tiab]) OR "psychological burnout"[tiab] OR "burnout"[tiab] OR "burnouts"[tiab]) OR "psychologic*"[tiab] | 1787854 |
| #4 | #1 AND #2 AND #3 | 920 |

**Table S2. Selection strategy in Embase**

| Search | Query | Items found |
| --- | --- | --- |
| #1 | ('neoplasm'/exp OR neoplasm:ab,ti OR 'cancer'/exp OR cancer:ab,ti OR 'tumor'/exp OR tumor:ab,ti OR 'tumour'/exp OR tumour:ab,ti) ('neoplasm'/exp OR neoplasm OR 'cancer'/exp OR cancer OR 'tumor'/exp OR tumor OR 'tumour'/exp OR tumour) | 5042206 |
| #2 | ('covid 19'/exp OR 'covid 19':ab,ti OR 'sars cov 2'/exp OR 'sars cov 2':ab,ti OR 'severe acute respiratory syndrome coronavirus 2'/exp OR 'severe acute respiratory syndrome coronavirus 2':ab,ti) | 185505 |
| #3 | ('depression'/exp OR depression:ab,ti OR 'distress'/exp OR distress:ab,ti OR 'stress'/exp OR stress:ab,ti OR 'anxiety'/exp OR anxiety:ab,ti OR 'post-traumatic stress symptoms':ab,ti OR 'posttraumatic stress disorder'/exp OR 'posttraumatic stress disorder':ab,ti OR 'ptsd'/exp OR ptsd:ab,ti OR 'burnout'/exp OR burnout:ab,ti) | 1755177 |
| #4 | #1 AND #2 AND #3 | 1671 |

**Table S3. Selection strategy in** **PsyclNFO**

| Search | Query | Items found |
| --- | --- | --- |
| #1 | mainsubject(neoplasm) OR noft(neoplasm) OR noft(cancer) OR noft(tumor) OR noft(tumour) | 108793 |
| #2 | noft(depression) OR noft(distress) OR noft(stress) OR noft(anxiety) OR noft("post-traumatic stress symptoms") OR noft("posttraumatic stress disorder") OR noft(PTSD) OR noft(burnout)) | 772417 |
| #3 | mainsubject(COVID-19) OR noft(COVID-19) OR noft(SARS-CoV-2) OR (noft(severe acute respiratory syndrome coronavirus 2)) | 12791 |
| #4 | #1 AND #2 AND #3 | 88 |

**Table S4. Selection strategy in Web of Science**

| Search | Query | Items found |
| --- | --- | --- |
| #1 | neoplasm OR cancer OR tumor OR tumour (All Fields) | 4781370 |
| #2 | COVID-19 OR SARS-CoV-2 OR (severe acute respiratory syndrome coronavirus 2) (All Fields) | 170760 |
| #3 | depression OR distress OR stress OR anxiety OR "post-traumatic stress symptoms" OR "posttraumatic stress disorder" OR PTSD OR burnout OR psychologic* (All Fields) | 2738864 |
| #4 | #1 AND #2 AND #3 | 1433 |

**Table S5 Summary of Psychological health status**

| **Study** | **Area** | **n** | **Case** | **Scale** | **Cut-off** | **Type of Cancer** |
| --- | --- | --- | --- | --- | --- | --- |
| **Depression** |  |  |  |  |  |  |
| Alrubai, T 2021 | Iraq | 200 | 44 | DASS-21 | ≥10 | Mixed |
| Arrieta, O 2021 | Mexico | 144 | 44 | DASS-21 | ≥5 | Thoracic |
| Bafunno, D 2021 | Italy | 175 | 77 | HADS-D | ≥8 | Mixed |
| Bao, M 2021 | China | 3197 | 347 | PHQ-9 | ≥10 | Hematological |
| Bauerle, A 2021 | Germany | 150 | 25 | PHQ-2 | ≥3 | Mixed |
| Chen, GL 2020 | China | 326 | 243 | SDS | ≥53 | Mixed |
| Chen, X 2021 | China | 834 | 180 | PHQ-9 | ≥10 | Breast |
| Faro, JM 2021 | USA | 61 | 16 | PHQ-2 | ≥3 | NR |
| Forner, D 2021 | Canada | 14 | 6 | HADS-D | ≥8 | Mixed |
| Frey, MK 2020 | USA | 555 | 147 | HADS-D | ≥8 | Gynaecological |
| Gultekin, M 2021 | Europe | 1251 | 641 | HADS-D | ≥8 | Gynaecological |
| Hu, L 2020 | China | 156 | 27 | PHQ-9 | ≥10 | Mixed |
| Jacobson, C 2021 | UK | 112 | 48 | PHQ-8 | ≥10 | Mixed |
| Juanjuan, L 2020 | China | 658 | 145 | PHQ-9 | ≥10 | Breast |
| Levy, I 2021 | Israel | 408 | 172 | PHQ-9 | ≥10 | Hematological |
| Lou, SC 2020 | China | 58 | 58 | SDS | ≥53 | Head and neck |
| Massicotte, V 2021 | Canada | 36 | 6 | HADS-D | ≥7 | Breast |
| Nardone, V 2021 | Italy | 78 | 43 | BDI-2 | ≥14 | Mixed |
| Ng, DWL 2020 | HongKong | 72 | 7 | HADS-D | ≥8 | Breast |
| Rodrigues-Oliveira, L 2021 | Brazil | 50 | 11 | HADS-D | ≥8 | Head and neck |
| Romito, F 2020 | Italy | 77 | 24 | HADS-D | ≥8 | Hematological |
| Toquero, P 2021 | Spain | 104 | 61 | HADS-D | ≥4 | Mixed |
| Turgeman, I 2021 | Israel | 164 | 39 | HADS-D | ≥8 | Mixed |
| van de Poll-Franse, LV 2021 | Netherlands | 4094 | 406 | HADS-D | ≥8 | NR |
| Wang, Y 2020 | China | 6213 | 1456 | PHQ-9 | ≥7 | Mixed |
| Wong, LP 2021 | Malaysia | 631 | 129 | HADS-D | >8 | Mixed |
| Yang, L 2021 | China | 373 | 126 | SDS | ≥53 | Mixed |
| Yang, SJ 2021 | China | 219 | 74 | PHQ-9 | ≥5 | Thyroid |
| **Anxiety** |  |  |  |  |  |  |
| Alrubai, T 2021 | Iraq | 200 | 44 | DASS-21 | ≥8 | Mixed |
| Arrieta, O 2021 | Mexico | 144 | 26 | DASS-21 | ≥4 | Thoracic |
| Bafunno, D 2021 | Italy | 173 | 101 | HADS-A | ≥8 | Mixed |
| Bao, M 2021 | China | 3197 | 226 | GAD-7 | ≥10 | Hematological |
| Bauerle, A 2021 | Germany | 150 | 31 | GAD-2 | ≥3 | Mixed |
| Chen, GL 2020 | China | 326 | 220 | SAS | ≥50 | Mixed |
| Chen, X 2021 | China | 834 | 129 | GAD-7 | ≥10 | Breast |
| Ellehuus, C 2021 | Denmark | 2205 | 457 | GAD-7 | ≥5 | Hematological |
| Faro, JM 2021 | USA | 61 | 15 | GAD-2 | ≥3 | NR |
| Forner, D 2021 | Canada | 14 | 9 | HADS-A | ≥8 | Mixed |
| Frey, MK 2020 | USA | 555 | 285 | HADS-A | ≥8 | Gynaecological |
| Guc, ZG 2021 | Turkey | 761 | 336 | STAI-S | ≥44 | Mixed |
| Gultekin, M 2021 | Europe | 1251 | 743 | HADS-A | ≥8 | Gynaecological |
| Hu, L 2020 | China | 156 | 20 | GAD-7 | ≥10 | Mixed |
| Jacobson, C 2021 | UK | 112 | 42 | GAD-7 | ≥10 | Mixed |
| Juanjuan, L 2020 | China | 658 | 147 | GAD-7 | ≥10 | Breast |
| Kamposioras, K 2020 | UK | 143 | 7 | GAD-7 | ≥10 | Colorectal |
| Lou, SC 2020 | China | 58 | 57 | SAS | ≥50 | Head and neck |
| Massicotte, V 2021 | Canada | 36 | 16 | HADS-A | ≥7 | Breast |
| Nardone, V 2021 | Italy | 78 | 49 | STAI-S | ≥40 | Mixed |
| Ng, DWL 2020 | HongKong | 72 | 6 | HADS-A | ≥8 | Breast |
| Ng, KYY 2020 | Singapore | 624 | 119 | GAD-7 | ≥10 | Mixed |
| Rodrigues-Oliveira, L 2021 | Brazil | 50 | 11 | HADS-A | ≥8 | Head and neck |
| Romito, F 2020 | Italy | 77 | 28 | HADS-A | ≥8 | Hematological |
| Toquero, P 2021 | Spain | 104 | 44 | HADS-A | ≥7 | Mixed |
| Turgeman, I 2021 | Israel | 164 | 17 | HADS-A | ≥8 | Mixed |
| van de Poll-Franse, LV 2021 | Netherlands | 4094 | 486 | HADS-A | ≥8 | NR |
| Wang, Y 2020 | China | 6213 | 1101 | GAD-7 | ≥7 | Mixed |
| Wong, LP 2021 | Malaysia | 631 | 183 | HADS-A | >8 | Mixed |
| Yang, L 2021 | China | 373 | 130 | SAS | ≥50 | Mixed |
| Yang, SJ 2021 | China | 219 | 87 | GAD-7 | ≥5 | Thyroid |
| Yang, SL 2021 | China | 609 | 86 | SAS | ≥50 | NR |
| Yang, SM 2020 | China | 1106 | 367 | SAS | ≥50 | Hematological |
| Yasin, AI 2021 | Turkey | 298 | 144 | STAI-S | ≥40 | Breast |
| **PTSD** |  |  |  |  |  |  |
| Bafunno, D 2021 | Italy | 169 | 81 | IES-R | ≥24 | Mixed |
| Bao, M 2021 | China | 3197 | 450 | IES-R | ≥26 | Hematological |
| Borsari, S 2021 | Italy | 355 | 115 | IES-R | ≥33 | Skin |
| Joly, F 2021 | France | 563 | 120 | IES-R | ≥33 | Mixed |
| Juanjuan, L 2020 | China | 658 | 344 | IES-R | ≥33 | Breast |
| Romito, F 2020 | Italy | 77 | 28 | IES-R | ≥24 | Hematological |
| Wang, Y 2020 | China | 6213 | 578 | IES-R | ≥33 | Mixed |
| Yang, SJ 2021 | China | 219 | 44 | IES-R | ≥24 | Thyroid |
| **Distress** |  |  |  |  |  |  |
| Bauerle, A 2021 | Germany | 150 | 82 | DT | ≥5 | Mixed |
| Mendonca, AB 2021 | Brazil | 91 | 45 | DT | ≥4 | Mixed |
| Nardone, V 2021 | Italy | 78 | 51 | DT | ≥4 | Mixed |
| Rades, D 2020 | Germany | 338 | 156 | DT | ≥5 | Breast |
| Rodrigues-Oliveira, L 2021 | Brazil | 50 | 29 | DT | ≥4 | Head and neck |
| **Insomnia** |  |  |  |  |  |  |
| Chen, X 2021 | China | 834 | 123 | ISI | ≥15 | Breast |
| Joly, F 2021 | France | 563 | 135 | ISI | ≥15 | Mixed |
| Juanjuan, L 2020 | China | 658 | 111 | ISI | ≥15 | Breast |
| Massicotte, V 2021 | Canada | 36 | 15 | ISI | ≥8 | Breast |
| Yang, SJ 2021 | China | 219 | 69 | ISI | ≥8 | Thyroid |
| **Fear** |  |  |  |  |  |  |
| Chen, GL 2020 | China | 326 | 282 | FoP-Q-SF | ≥34 | Mixed |
| Massicotte, V 2021 | Canada | 36 | 19 | FCRI | ≥13 | Breast |
| Soriano, EC 2021 | USA | 50 | 30 | FCRI | ≥13 | Breast |

DASS-21, Depression, Anxiety, and Stress Scale; PHQ-2, the 2-item Patient Health Questionnaire; PHQ-8, the 8-item Patient Health Questionnaire; PHQ-9, the 9-item Patient Health Questionnaire; GAD-2, the 2-item Generalized Anxiety Disorder; GAD-7, the 7-item Generalized Anxiety Disorder; IES-R, the 22-item Impact of Event Scale-Revised; ISI, the Insomnia Severity Index; DT, Distress thermometer; FoP-Q-SF, the Fear of Progression Questionnaire-Short Form; STAI-S, the State-Trait Anxiety Inventory (State); BDI-2, the Beck Depression Inventory vers.2; HADS, Hospital Anxiety and Depression Scale; FCRI, the Fear of Cancer Recurrence Inventory; SAS, Self-Rating Anxiety Scale; SDS, Self-Rating Depression Scale.

**Table S6. Risk of bias in the included studies**

| **Study** | **Item 1** | **Item 2** | **Item 3** | **Item 4** | **Item 5** | **Item 6** | **Item 7** | **Item 8** | **Item 9** | **Overall** |
| --- | --- | --- | --- | --- | --- | --- | --- | --- | --- | --- |
| Alrubai, T 2021 | Unclear | Low | Unclear | Low | Low | Low | Low | Low | Low | Low |
| Arrieta, O 2021 | Low | Low | Unclear | Unclear | Low | Low | Low | Low | Low | Low |
| Bafunno, D 2021 | Unclear | Low | Unclear | Low | Low | Low | Low | Low | Low | Low |
| Bao, M 2021 | Unclear | Unclear | Low | Low | Low | Low | Low | Low | Low | Low |
| Bauerle, A 2021 | Unclear | Unclear | Unclear | Low | Low | Low | Low | Low | Low | Unclear |
| Borsari, S 2021 | Unclear | Low | Unclear | Low | Low | Low | Low | Low | High | High |
| Chen, GL 2020 | Unclear | Unclear | Unclear | Low | Low | Low | Low | Low | Low | Unclear |
| Chen, X 2021 | Low | Low | Low | Low | Low | Low | Low | Low | Low | Low |
| Ellehuus, C 2021 | Unclear | Low | Low | Low | Low | Low | Low | Low | High | High |
| Faro, JM 2021 | Unclear | Unclear | Unclear | Low | Low | Low | Low | Low | Low | Unclear |
| Forner, D 2021 | Unclear | Low | Unclear | Low | Low | Low | Low | Low | High | High |
| Frey, MK 2020 | Low | Unclear | Low | Unclear | Low | Low | Low | Low | Low | Low |
| Guc, ZG 2021 | Unclear | Low | Low | Low | Low | Low | Low | Low | High | High |
| Gultekin, M 2021 | Unclear | Unclear | Low | Unclear | Low | Low | Low | Low | Low | Unclear |
| Hu, L 2020 | Unclear | Unclear | Unclear | Low | Low | Low | Low | Low | Low | Unclear |
| Jacobson, C 2021 | Unclear | Low | Unclear | Unclear | Low | Low | Low | Low | High | High |
| Joly, F 2021 | Unclear | Unclear | Low | Unclear | Low | Low | Low | Low | Low | Unclear |
| Juanjuan, L 2020 | Low | Unclear | Low | Low | Low | Low | Low | Low | Low | Low |
| Kamposioras, K 2020 | Unclear | Low | Unclear | Unclear | Low | Low | Low | Low | High | High |
| Levy, I 2021 | Unclear | Unclear | Unclear | Low | Low | Low | Low | Low | High | High |
| Lou, SC 2020 | Low | Unclear | Unclear | Low | Low | Low | Low | Low | Low | Low |
| Massicotte, V 2021 | Low | Low | Unclear | Low | Low | Low | Low | Low | High | High |
| Mendonca, AB 2021 | Unclear | Unclear | Low | Unclear | Low | Low | Low | Low | Low | Unclear |
| Nardone, V 2021 | Unclear | Unclear | Unclear | Unclear | Low | Low | Low | Low | Low | Unclear |
| Ng, DWL 2020 | Unclear | Unclear | Unclear | Low | Low | Low | Low | Low | Low | Unclear |
| Ng, KYY 2020 | Unclear | Unclear | Low | Low | Low | Low | Low | Low | Low | Low |
| Rades, D 2020 | Unclear | Unclear | Unclear | Unclear | Low | Low | Low | Low | Low | Unclear |
| Rodrigues-Oliveira, L 2021 | Low | Low | Low | Low | Low | Low | Low | Low | Low | Low |
| Romito, F 2020 | Unclear | Low | Unclear | Low | Low | Low | Low | Low | Low | Low |
| Soriano, EC 2021 | Low | Unclear | Unclear | Low | Low | Low | Low | Low | High | High |
| Toquero, P 2021 | Unclear | Low | Unclear | Low | Low | Low | Low | Low | Low | Low |
| Turgeman, I 2021 | Unclear | Unclear | Unclear | Unclear | Low | Low | Low | Low | Low | Unclear |
| van de Poll-Franse, LV 2021 | Unclear | Unclear | Low | Unclear | Low | Low | Low | Low | High | High |
| Wang, Y 2020 | Unclear | Unclear | Low | Low | Low | Low | Low | Low | Low | Low |
| Wong, LP 2021 | Unclear | Unclear | Low | Unclear | Low | Low | Low | Low | Low | Unclear |
| Yang, L 2021 | Unclear | Low | Unclear | Low | Low | Low | Low | Low | Low | Low |
| Yang, SJ 2021 | Unclear | Unclear | Unclear | Low | Low | Low | Low | Low | Low | Unclear |
| Yang, SL 2021 | Unclear | Unclear | Low | Unclear | Low | Low | Low | Low | Low | Unclear |
| Yang, SM 2020 | Low | Unclear | Low | Unclear | Low | Low | Low | Low | Low | Low |
| Yasin, AI 2021 | Low | Low | Unclear | Low | Low | Low | Low | Low | High | High |

Item 1, Was the sample frame appropriate to address the target population?

Item 2, Were study participants sampled in an appropriate way?

Item 3, Was the sample size adequate?

Item 4, Were the study subjects and the setting described in detail?

Item 5, Was the data analysis conducted with sufficient coverage of the identified sample?

Item 6, Were valid methods used for the identification of the condition?

Item 7, Was the condition measured in a standard, reliable way for all participants?

Item 8, Was there appropriate statistical analysis?

Item 9, Was the response rate adequate, and if not, was the low response rate managed appropriately?
